# High-contrast en bloc staining of mouse whole-brain and human brain samples for EM-based connectomics

**DOI:** 10.1038/s41592-023-01866-3

**Published:** 2023-05-08

**Authors:** Kun Song, Zhihui Feng, Moritz Helmstaedter

**Affiliations:** Department of Connectomics, Max Planck Institute for Brain Research, Frankfurt, Germany

**Keywords:** Cellular neuroscience, Synaptic transmission, Mouse, Transmission electron microscopy

## Abstract

Connectomes of human cortical gray matter require high-contrast homogeneously stained samples sized at least 2 mm on a side, and a mouse whole-brain connectome requires samples sized at least 5–10 mm on a side. Here we report en bloc staining and embedding protocols for these and other applications, removing a key obstacle for connectomic analyses at the mammalian whole-brain level.

## Abstract (web summary)

For EM-based connectomics applications, a staining protocol for large tissue samples in the range of a centimeter has been developed, which avoids artifacts common with established protocols.

## Main

**Fig. 1:**
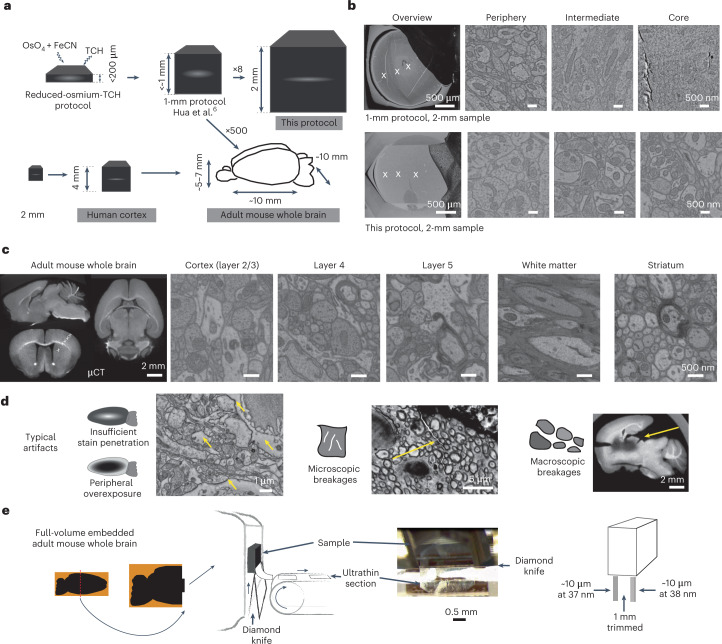
High-contrast staining protocol for brain samples 2 mm to 1 centimeter in size, applied to human cortical tissue and adult mouse whole brains. **a**, Overview of en bloc staining protocol development: reduced-osmium-TCH protocols^1–3,25^ for samples up to about 200–250 μm in thickness and a more recent^6^ 1-mm protocol, compared to the protocol reported here (gray boxes) enabling gradient-free en bloc staining of samples 2 mm to 1 centimeter in size (sufficient to cover the depth of the human cortex (Fig. 2c–e) and entire adult mouse brains). **b**, Staining of a sample 2 mm in size using the 1-mm protocol^6^ (top) and our protocol (bottom) (SEM images at a voxel size of 11.24 nm; dose, 47 e^−^ nm^−2^). **c**, High-contrast staining of whole mouse brains. High-resolution EM images acquired from the superficial cortex to subcortical tissue; approximate positions are indicated in the μCT overview (left). High-resolution SEM images (at a voxel size of 5.62 × 5.62 nm^2^; 113 e^−^ nm^−2^). μCT data of whole brains: https://wklink.org/7742 and https://wklink.org/7789. **d**, Illustrations and examples of key challenges of hemisphere and mouse whole-brain staining. Yellow arrows point to sites of intracellular overextraction with loss of cytosolic components (left) and breakages of the sample at microscopic (middle) or macroscopic (right) scale. **e**, ATUM cutting test at the center of a fully stained and fully embedded mouse whole-brain sample (sample W3, Spurr’s resin, Supplementary Table 1). Middle, sketch and picture of ATUM cutting at sample center, trimmed to about 4 × 2 mm^2^ for ATUM cutting. Right, sketch of the location of two artifact-free ATUM test series at the exposed surface and after an additional depth of 1 mm (Supplementary Video 1).

The dense and homogeneous deposition of heavy metals into brain tissue that leads to high membrane contrast for electron-based imaging is a prerequisite for synaptic-resolution connectomics. Ever since the development of ‘reduced-osmium’ protocols^1–3^, en bloc staining of tissue samples up to a thickness of about 100–200 µm was possible. Beyond such sample sizes, however, substantial staining gradients occurred, which limited connectomic analyses to smaller samples^4,5^. With the development of a modified staining protocol^6^, samples up to about 1 mm in size could be homogeneously stained, an important step to allow millimeter-size connectomic data acquisition (Fig. 1a). This protocol has been widely applied since^7–10^.

However, with the ambition to obtain connectomes from even larger samples, in particular, samples that encompass the gray matter depth of the human cortex (at least 2 mm in size) and samples corresponding to entire brains of small mammals such as mice^11^ or even humans^12^, the need for improved protocols became obvious. In spite of the promising initial attempts for mouse whole-brain staining^13^, there is thus far no reliable protocol for en bloc staining of multi-mm-to-centimeter-scale samples with high staining contrast. This is particularly challenging, as EM imaging and reconstruction have progressed to a stage at which large-sample analyses appear to become possible^7,8,14^.

**Fig. 2:**
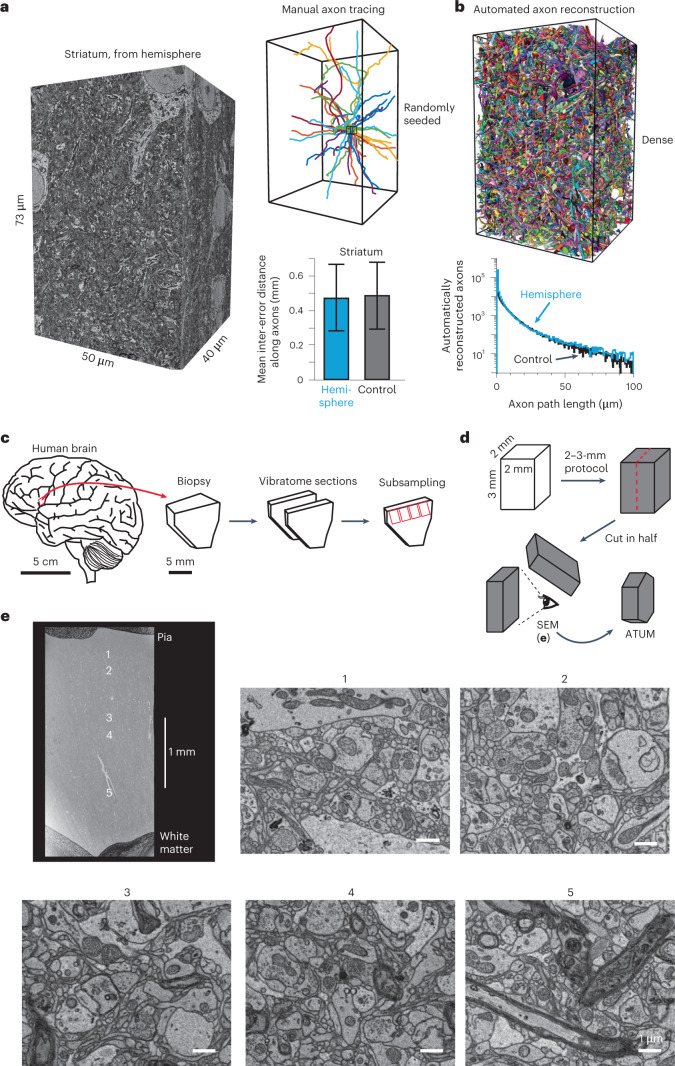
Traceability assessment in a mouse hemisphere sample and application to the human cortex. **a**, SBEM dataset (pixel size, 11.24 × 11.24 nm^2^; cutting thickness, 35 nm; electron dose, 28 e^−^ nm^−2^) from the center of a fully stained and fully embedded mouse hemisphere (H6), located in the striatum. Twenty randomly seeded axons (from a bounding box 3 × 3 × 3 μm in size) manually reconstructed in the entire dataset. Control SBEM dataset of a mouse striatum from a conventional sample 1 × 1 × 0.5 mm^3^ in size stained using the 1-mm protocol^6^ and analyzed in the same way (gray bar), yielding a similar mean inter-error distance for axon reconstruction (mean and 25th and 75th percentiles; Kolmogorov–Smirnov test, *P* = 0.4814; Methods). **b**, Automated dense axon reconstruction (method as in ref. ^24^) with resulting axon path-length distributions (bottom) for hemisphere striatum (blue) and control striatum (black) datasets. **c**, Immersion fixation, vibratome slicing and sample generation from human brain biopsies from neurosurgical samples (same procedure as in ref. ^24^). **d**, Processing of a human brain sample using this protocol for large-volume staining (here, a 2 × 2 × 3-mm block = 12 mm^3^). **e**, Overview EM of a tissue block 3 mm in height spanning the pia and layers 1–6 of the human cortex (top left); high-resolution EM images of various locations along the cortical axis (right and bottom). Pixel size in **e**, (11.24 nm)^2^; electron dose, 42 e^−^ nm^−2^.

Here, we report such protocols for samples 2 mm in size (Fig. 1a,b, 8 mm^3^), mouse hemispheres and whole brains (Fig. 1a,c, 250–500 mm^3^; see ref. ^15^ for an initial version of our protocol) and human cortex samples (Fig. 2c–e, 12 mm^3^). Their development required the concomitant solution of the following problems: recurring staining inhomogeneity, sample instability that leads to breakages (especially in hemispheres and whole-brain samples) and inhomogeneous resin infiltration, as described in the following.

**Extended Data Fig. 1:**
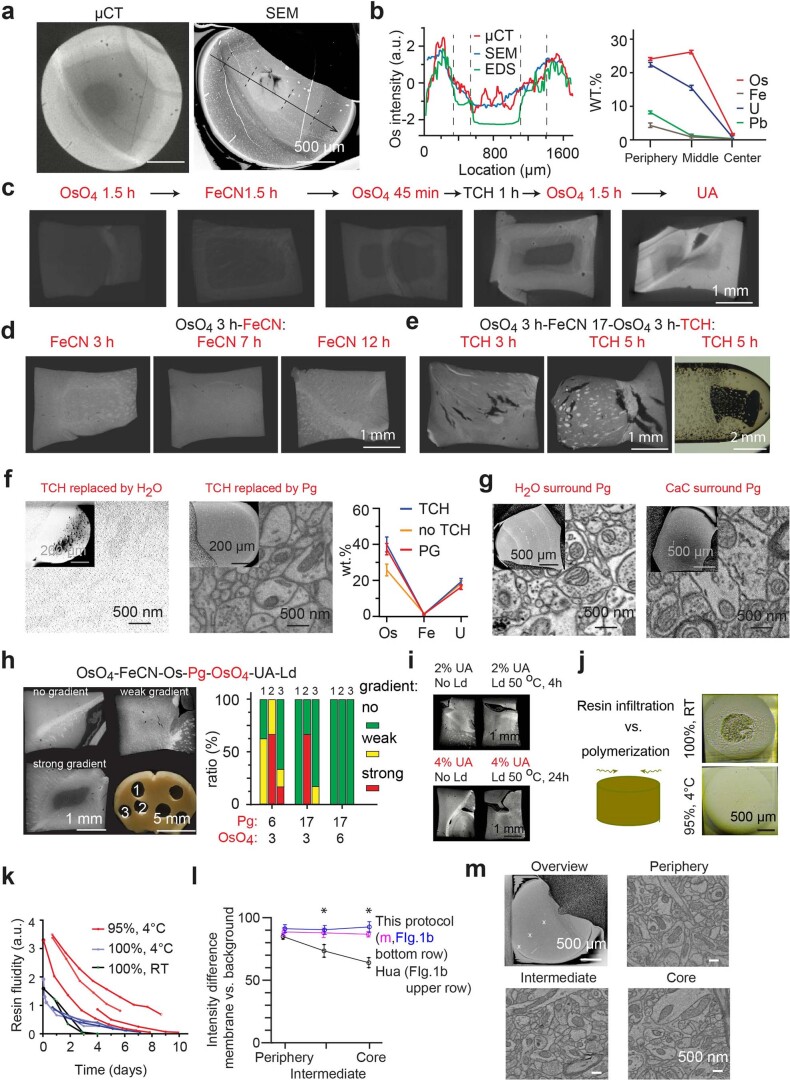
Development of 2 mm protocol. **(a)** Left: µCT image of staining gradient produced on 2 mm samples with Hua protocol^1^ and confirmation with SEM (right). **(b)** EDS analysis of staining gradient from sample in (a). **(c)** µCT diagnosis of staining gradient after each consecutive step of the Hua staining protocol^1^. **(d)** µCT images after extension of FeCN step from 3 h to 12 h. **(e)**. µCT images after extending TCH incubation. **(f)** Comparison of conductivity and membrane contrast of 1 mm samples stained with TCH vs Pyrogallol. (left) samples stained by 1 mm Hua protocol but with omission of TCH step; (middle) samples stained by 1 mm protocol but replacing TCH by pyrogallol;^2^ (right) EDS analysis of the final osmium concentration in the corresponding samples. **(g)**. Contrast and gradient comparison of pyrogallol-related steps in water (top) vs. in CaC (bottom). Electron dose for high resolution EM images in f,g,i:18 e^−^/nm^2^**. (h)** Effect of duration of pyrogallol related steps. (left) influence of sampling location on the pyrogallol related gradient; (right) extension of pyrogallol incubation and 3^rd^ OsO_4_ step removed gradient for all sampling locations. **(i)** Gradients related to UA and Ld incubation under different concentrations (UA only) and incubation time. **(j,k)** Resin infiltration speed in dependence of temperature and acetone concentration. Resin infiltration of larger samples requires separation of diffusion from polymerization to avoid softer core of sample (j).The resin fluidity time^−^course curve (k) was measured for different conditions of temperature and acetone addition combination (see Methods). **(l)**. Quantification of membrane contrast (see Methods) in dependence of location within sample for Hua protocol (1b upper), our 2 mm protocol-Os3 (Extended Data Fig. 1m) and Os24 (1b lower). **(m)** EM screening results for our 2 mm protocol-Os3.

**Extended Data Fig. 2:**
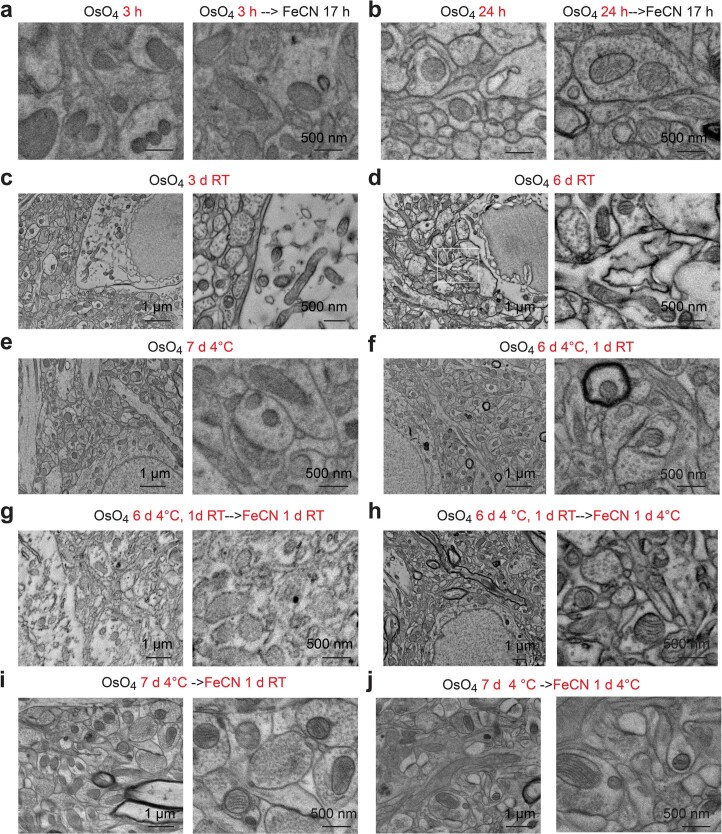
Effect of Os and FeCN incubation time and temperature on membrane contrast in 2 mm samples. **(a)** Membrane contrast comparison of samples stained with 3 h of OsO_4_ (left) or with 3 h OsO_4_ followed by 17 h of FeCN. **(b)** Membrane contrast comparison of samples stained with 24 h of OsO_4_ (left), or with 24 h of OsO_4_ followed by 17 h of FeCN. **(c,d)** Ultrastructural quality of samples stained with 3 (c) and 6 (d) days of OsO_4_ at room temperature. **(e,f)** Ultrastructural quality of samples stained with 7d incubation of OsO_4_ at 4 °C (e) or with 6d incubation of OsO_4_ at 4 °C followed by 1 d incubation at RT (f). **(g-j)**. Membrane contrast and ultrastructural quality of samples stained with OsO_4_ and FeCN under different incubation time and temperature: (g) 6d incubation of OsO_4_ at 4 °C followed by 1 d incubation at RT, plus 1d FeCN incubation at RT; (h) 6d incubation of OsO_4_ at 4 °C followed by 1d incubation at RT, plus 1d FeCN incubation at 4 °C; (i) 7d with OsO_4_ at 4 °C followed by 1d of FeCN at RT; (j) 7d with OsO_4_ at 4 °C followed by 1d of FeCN at 4 °C. All images were acquired in low vacuum SEM at 30 Pa, with maximum electron dose 70 e^−^/nm^2^.

For protocol development, we initially used X-ray microtomography (µCT) imaging to assess staining gradients^13,16^. Additionally, we applied low-vacuum scanning electron microscopy (SEM)^17,18^ to evaluate ultrastructural contrast. This was of particular importance, as samples that had a homogeneous appearance in µCT could reveal insufficient membrane contrast or damaged ultrastructure when analyzed by electron microscopy (EM) (Extended Data Figs. 1 and 2).

To achieve the ultimate goal of a 500-fold enlarged infiltration volume (mouse whole brain versus cubic millimeter, Fig. 1a), we first worked on the staining of a volume about eightfold to tenfold larger (‘2-mm’ samples, Fig. 1a,b). When we applied the available 1-mm protocol^6^ to samples 2 mm in size (Fig. 1b), we observed strong staining gradients (Fig. 1a and Extended Data Fig. 1a,b) and incomplete resin infiltration (Extended Data Fig. 1j). We then used µCT to investigate at which steps in the protocol the gradients occurred, analyzed their co-dependence and found that, by prolonging relevant incubation steps, targeted separation from washing steps, replacing thiocarbohydrazide (TCH) with pyrogallol (Pg) as proposed in ref. ^13^ and introducing additional steps to ensure sample stability, we could obtain gradient-free high-contrast samples sized about 2 mm on a side (Fig. 1b, bottom; see Supplementary Results for a more detailed description of the protocol development and insights from these investigations). For obtaining homogeneous resin infiltration, we considered the fact that the epoxy resin blender would undergo polymerization during infiltration, which would increase the viscosity of the blender, and, once the polymerization process has crossed the gel point, no more diffusion would be possible^19^. Thus, the practical strategy for improving resin infiltration was to slow down polymerization reactions and maintain low viscosity. To do so, we kept all resin-infiltration steps at 4 °C^19,20^, added a step with 95% resin in 5% acetone (this small amount of acetone substantially decreases the resin viscosity^21^) and extended the incubation times (for details, see Supplementary Table 1). By these modifications, we infiltrated both Spurr’s and Epon epoxy resin into the sample center, yielding homogeneously embedded samples 2 mm in size (Extended Data Fig. 1j,k).

**Extended Data Fig. 3:**
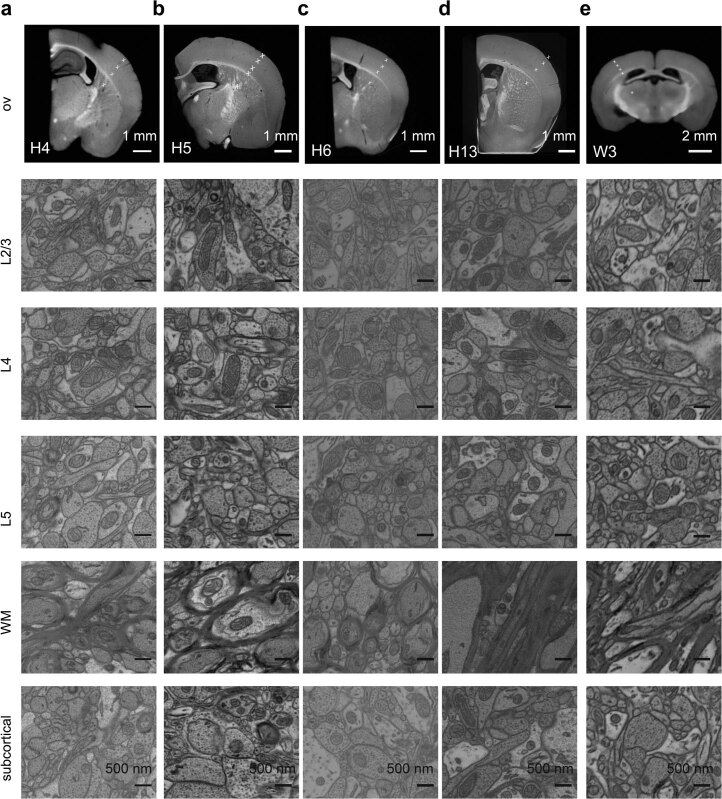
Reproduction of mouse hemisphere stainings. **(a-d)** Hemispheres H4-H6 (in total n = 4 successful replicates) were stained as in Supplementary Fig. 7, Supplementary Table 3. **(e)** Whole brain W3 was stained as in Extended Data Fig. 7, Supplementary Table 3. Together with W1 (Fig. 1c), a total of n = 2 whole brains were stained with parameters reported in Extended Data Fig. 7, Supplementary Table 3. All SEM images were taken at high vacuum with 5.62 nm pixel size and a dose of 113 e^−^/nm^2^.

**Extended Data Fig. 4:**
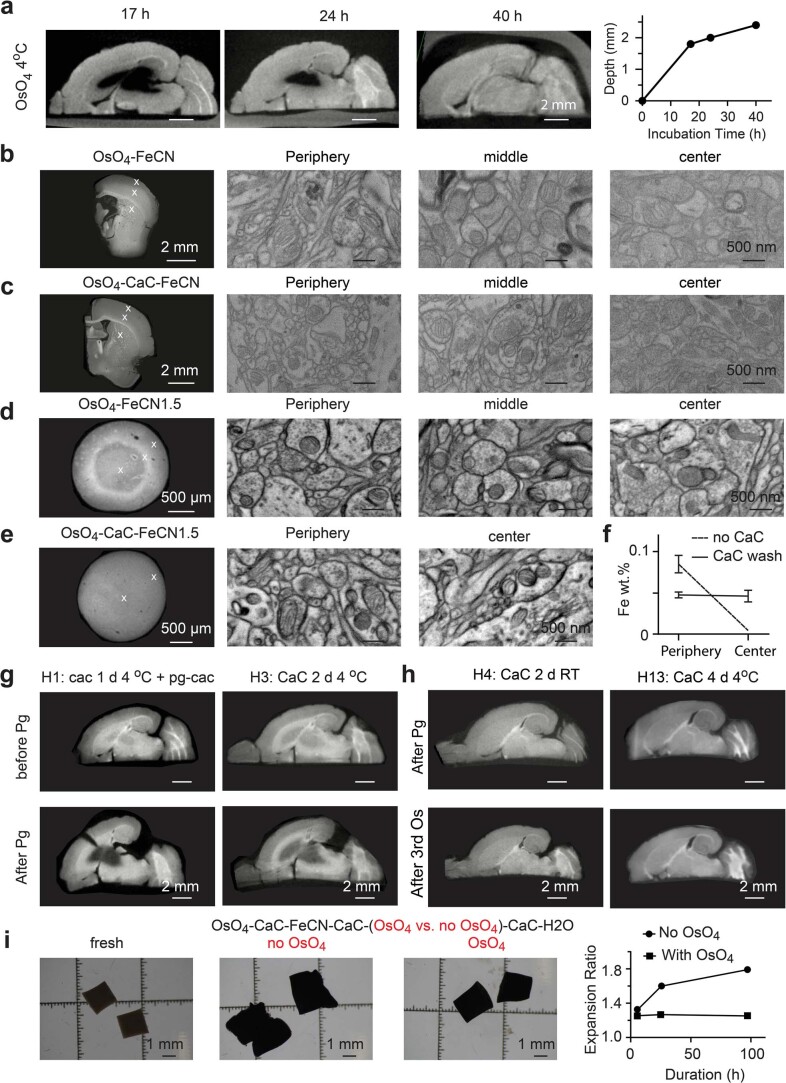
Gradients and breakages in mouse hemispheres. a Observation of OsO_4_ diffusion into mouse hemisphere sample at successive time points at 4 °C using µCT. **(b,c)** Effect of buffer rinsing between OsO_4_ and FeCN incubation on staining gradient. **(d,e)** Effect of buffer rinsing between OsO_4_ and FeCN incubation on FeCN diffusion speed and membrane contrast. **f** EDS measurement of Fe concentration in the center of samples from d,e. **g** gradient (top) after insufficient buffer washing step (1 or 2 days) at 4 °C; gradient amplification and breakages occured in Pg (bottom). **h** No gradient after sufficient buffer washing (2 days RT or 4 days 4 °C). **i** Effect of adding an extra OsO_4_ incubation step after FeCN on the sample stability in H_2_O measured as sample expansion ratio. All SEM images were acquired in low vacuum SEM at 30 Pa, with maximum electron dose 70 e^−^/nm^2^.

**Extended Data Fig. 5:**
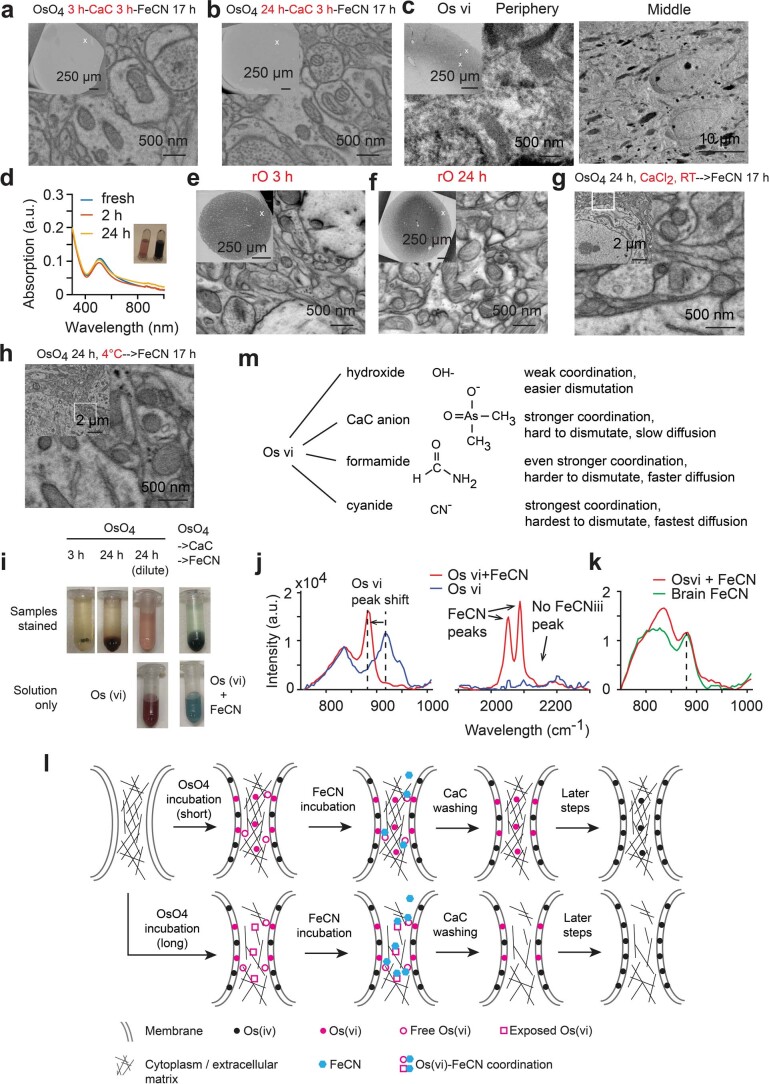
Experiments conducted for exploring possible chemical mechanisms underlying staining contrast generation. **(a,b)** Membrane contrast after adding an intermediate CaC washing step between OsO_4_ and FeCN. Imaged in SEM in high vacuum, electron dose 31 e^−^/nm^2^. **(c)** Membrane contrast after staining with potassium osmate (vi) (note yielded precipitation band). **(d)** UV-vis spectrum of potassium osmate (vi) in sodium cacodylate buffer over 24 h. **(e,f)** 2 mm samples stained with ‘reduced osmium’ protocol for 3 h vs. 24 h. **(g)** Membrane contrast after adding CaCl_2_ into OsO_4_ before FeCN incubation. **(h)** Membrane contrast after OsO_4_ incubation at 4 °C before FeCN incubation. SEM acquisition for panels c,e,f,g,h was performed in low vacuum at 30 Pa, maximum electron dose of 70 e^−^/nm^2^. **(i-m)** Possible chemical logic underlying staining results **(i)** Comparison of bright-field color appearance of samples stained in OsO_4_ for 2 h, 24 h, 24 h (diluted to evaluate color), and stained in OsO_4_(24 h)->CaC->FeCN (intermediate CaC wash to remove OsO_4_), top row. For comparison, pure staining solutions are shown below: Os(vi) and in vitro reaction of Os(vi) with FeCN (bottom). Note that sample solution after 24 h OsO_4_ incubation appears similarly colored as Os(vi) solution (pink); and sample solution after Os-Cac-FeCN staining appears similarly colored as Os(vi)+FeCN reaction in vitro (blue^−^green). **(j)**. (left) Raman spectrum measurements of the product of Os(vi) + FeCN showing a shifted Os(vi) peak; (middle) in the wavelength range corresponding to FeCN(ii) and FeCN(iii), there was only signal consistent with FeCN(ii) peaks, but not FeCN(iii), suggesting the reaction between Os(vi) and FeCN was not a redox reaction. **(k)**. The same Os(vi) signal was observed in brain staining solution after FeCN staining and in-vitro Os(vi) + FeCN reaction. **(l)** Sketch summary of the possible chemical reactions (see in Supplementary Results). **(m)** Summary of Os(vi) coordination chemistry potentially relevant for staining mechanisms.

To obtain staining and embedding protocols for mouse hemispheres about 25-fold larger (Extended Data Fig. 3) and adult mouse whole brains about 50-fold larger (Fig. 1c–e), we first extended the initial osmium tetroxide (OsO_4_) incubation to 3 d and 6 d, which resulted in increased membrane contrast, also in the sample center. But, we encountered a serious obstacle not found in smaller samples: when incubating for a sufficient duration for OsO_4_ to reach the center of the hemisphere sample, the outer parts of the sample were showing strong signs of ultrastructural disintegration (Fig. 1d; of note, these were not apparent in µCT, only under EM imaging). The cytosol of most neurites and cell bodies had an extracted appearance, possibly indicating the removal of intracellular proteins (Fig. 1d; note that Hayat^22^ had already discussed this in the context of overfixation of tissue by OsO_4_). As the outer part of the sample corresponds to the cortical gray matter, a key target of whole-brain connectomic analysis, whole-brain staining with good stain penetration but insufficient ultrastructural quality in the cortical periphery would be inadequate. Because the ultrastructural alterations were likely a result of protein overoxidization by prolonged OsO_4_ incubation (Supplementary Results), which could be slowed down with a lower incubation temperature (4 °C)^22^, we used lower temperatures for OsO_4_ and ferrocyanide (FeCN) incubation, which solved the peripheral ultrastructural disintegration problem. Additional challenges from the FeCN step were addressed by modified washing steps around the relevant incubations (Supplementary Results and Extended Data Figs. 4b–f and 5a,b).

**Extended Data Fig. 6:**
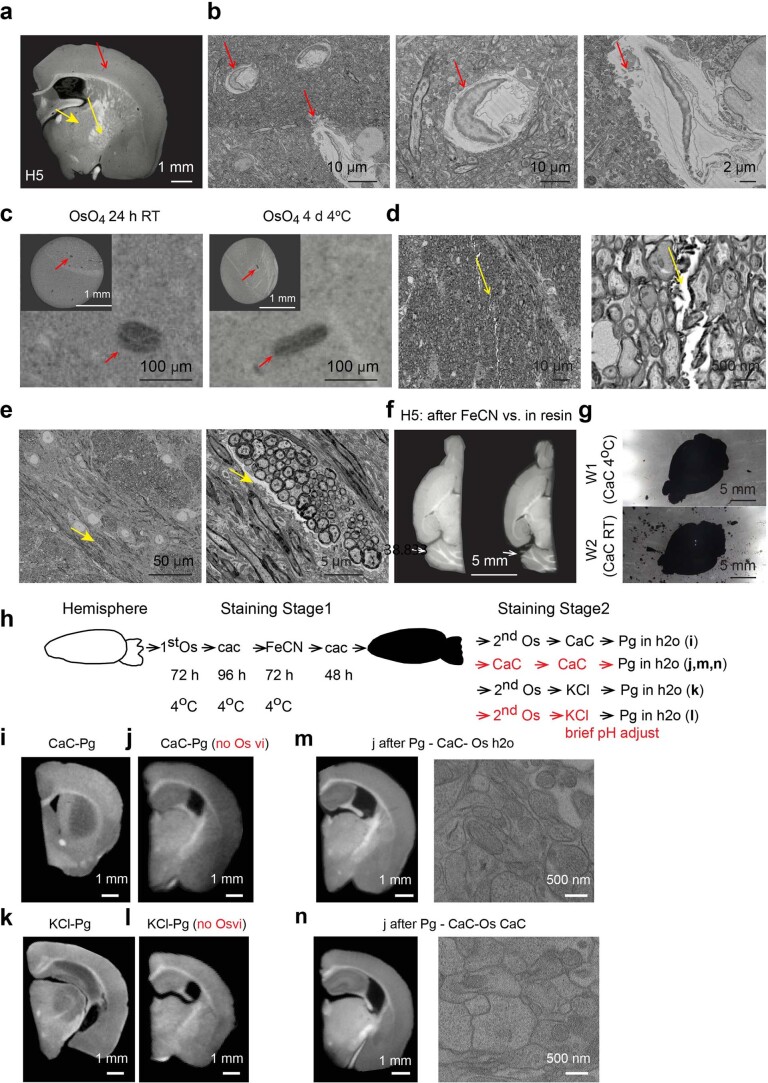
Summary of remaining artifacts in whole−brain staining protocol and alternative protocol approach for especially sensitive samples. **(a)** μCT cross section of H5 showing locations with artifacts detailed in b-e. **(b)** Red arrow: ‘Vessel artifact’: disruption of blood vessel pericytes from surrounding neuropil. **(c)** Vessel artifact visible in μCT images of 2 mm samples stained with OsO_4_ RT 1 day or 4 °C for 4 days. **(d,e)** Yellow arrows: Micro-breakages with width of less than 1 μm were occasionally observed in subcortical areas, in particular in highly myelinated regions. **(f)** During H_2_O incubation, the cerebellum was especially sensitive to macroscopic damage. **(g)** Changing the CaC rinsing step between 1^st^ OsO_4_ and FeCN to 4 °C improved the stability of the cerebellum for whole-brain staining. All SEM images were acquired at high vacuum, with maximum dose of 21 e^−^/nm^2^. **(h)**. Sketch of experiments performed to explore alternatives to incubation in plain H_2_O around Pg step, which may create high osmotic stress. **(i-l)** Influence of possible free Os (vi) in the sample on establishment of staining gradient during pyrogallol incubation (see Supplementary Results). **(m)** Samples from j further stained with Os in water resulted in acceptable membrane contrast. **(n)** Samples from j further stained with Os in CaC resulted in acceptable membrane contrast. **m,n** imaged under low-vacuum SEM (60 pa, beam current ~55 pA), pixel size 11.24 nm, electron dose ~25 e^−^/nm^2^.

A major remaining problem was that larger samples were consistently broken during the staining process, either in their entirety into several smaller pieces or with microbreakages that would hamper dense circuit reconstruction (Fig. 1d). These breakages would usually occur in relation to the Pg incubation step (Extended Data Fig. 4g,h). We therefore had to assess the effect of all earlier protocol steps on the stability of the sample during Pg incubation. This was of particular importance when the Pg incubation (including the washing steps before and after) was performed in water, as determined for the 2-mm samples (Extended Data Fig. 1g), and this water incubation could induce substantial osmotic forces in the sample. We found that inserting an additional extended OsO_4_ incubation step after the FeCN step would stabilize samples such that they could be incubated in water for up to 100 h without the occurrence of major breakages (Extended Data Fig. 4i; a possible explanation is the membrane perforation induced by OsO_4_, which increases tolerance to osmotic stress^22^). While this additional OsO_4_ step may also serve to enhance background staining and thus sample conductivity for SEM, in our view, the enhanced stability of large samples is the most critical aspect (see Extended Data Fig. 6h–n for an alternative approach to avoid incubation with pure water by omitting the second osmium step).

**Extended Data Fig. 7:**
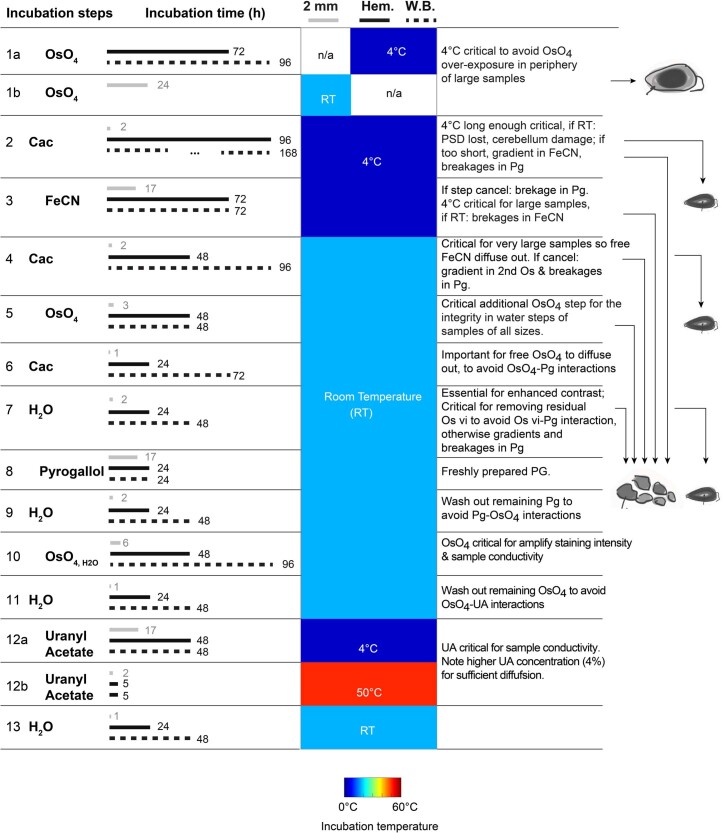
Overview of 2 mm, hemisphere and whole−brain protocol steps with incubation times and temperature steps visualized. Illustration of the relevance of various staining and washing steps for the key challenges of whole^−^brain staining: avoidance of gradients, peripheral tissue destruction, and macro- or microbreakages. Note for intermediate sized samples (larger than 2 mm in any dimension), incubation times should be increased by roughly interpolating to the hemisphere protocol.

We applied the hemisphere protocol to *n* = 4 hemispheres (Extended Data Fig. 3a–d), which all remained intact and provided homogeneous high-contrast staining throughout the sample. In addition, we stained two entire mouse brains with modest additional extensions of incubation times (Fig. 1c and Extended Data Fig. 3e), yielding a mouse whole-brain staining protocol for large-scale connectomics^11^ (Extended Data Fig. 7). We observed two types of artifacts that remained in large-sample staining (Extended Data Fig. 6): the detachment of larger blood vessel walls from the surrounding neuropil and rare remaining microbreakages in the subcortical regions with a high rate of myelinated fibers. Also, special care had to be taken to preserve the integrity of the cerebellum (Extended Data Fig. 6f,g). In general, we recommend using longer protocol durations when in doubt about which protocol version to choose (for example, use the whole-brain protocol for samples even slightly larger than a hemisphere).

To confirm that our whole-brain embedding protocol would also yield sufficient sample stability for ultrathin cutting, we performed a test series of automated tape-collecting ultramicrotome (ATUM) cutting^23^ on the center of a mouse whole-brain sample that was stained and embedded as a whole and only trimmed for the experiment afterward (Fig. 1e and Supplementary Video1).

To quantitatively determine the sufficiency of our staining quality and resin stability in the center of our samples for three-dimensional (3D) EM acquisition and axon reconstruction, we acquired a serial block-face SEM (SBEM) dataset from the center (striatum) of one stained and full-volume embedded mouse brain hemisphere (Extended Data Fig. 3c; H6, webKnossos link) and compared this to a control SBEM dataset from the striatum obtained from a sample 1 × 1 × 0.5 mm^3^ in size stained with the 1 mm protocol^6^. Both manual axon reconstruction (Fig. 2a) and automated axon reconstruction (Fig. 2b) yielded similar results between locally stained control and hemisphere-stained samples.

Finally, we applied our protocol to a sample of human brain biopsy to directly test the applicability of our protocol to large human brain samples (Fig. 2c–e; tissue was obtained as described in ref. ^24^, patient H6). We stained and embedded a sample 2 × 2 × 3 mm^3^ in size (that is, 12 mm^3^ in volume) spanning all cortical layers from the human frontal cortex. The tissue was homogeneously stained across layers (Fig. 2e). Furthermore, we obtained a cutting series using ATUM to test cuttability for large-scale 3D EM (2,733 slices at a cutting thickness of 35–40 nm from the sample center).

We envision that the protocols reported here will be useful for large-scale connectomic projects in mice and other species. In particular, the volume of a mouse brain also approaches relevant cortical and subcortical volumes in higher mammals such as non-human primates and humans. The goal of obtaining large connectomes from fractions or the entirety of the human cortex^12^ will also profit from the advances described here, both for fundamental research and possible clinical applications, such as connectomic analysis of pathological brain specimens from neurosurgical interventions^24^.

## Methods

### Animal experiments

All experimental procedures were approved by the local animal care and use committee and were in accordance with the laws of animal experimentation issued by the German federal government (Regierungspräsidium Darmstadt, Germany, permits V54, 19 c 20/15, F126/1028 and F126/1002).

Adult C57BL6/J mice (male and female, P30–P90) were treated with analgesics (0.1 mg per kg buprenorphin (CP-Pharma) and 100 mg per kg metamizol (WDT)) for 0.5 h before isoflurane anesthesia (Harvard Apparatus; 5% in O_2_ for initialization, 2–3% for maintenance; O_2_ flow rate, 1 l min^−1^). Following anesthesia, the animals were transcardially perfused (Harvard Apparatus, flow rate of 10 ml min^−1^) using 15 ml sodium cacodylate buffer (0.15 M, pH 7.4, Sigma-Aldrich) followed by 30 ml fixative containing 2.5% paraformaldehyde (Sigma-Aldrich), 1.25% glutaraldehyde (Serva) and 2 mM calcium chloride (Sigma-Aldrich) in 0.08 M sodium cacodylate buffer (osmolarity of about 700–800 mmol per kg, pH 7.4). The duration between the start of perfusion and incision of the diaphragm was less than 30 s. After perfusion, animals were decapitated, the skull was opened with care to avoid mechanical damage to the brain, and the brain was post-fixed in situ for 12–96 h at 4 °C before extraction from the skull.

For 2-mm samples, the brain was cut into 2-mm-thick coronal sections in 0.15 M cacodylate buffer using a vibratome (Leica, VT1200). Next, a biopsy punch 2 mm in diameter (Kai Medical) was used to extract samples from dorsal cortical, ventral cortical and subcortical regions (Supplementary Fig. 1h). The samples were then stored in 0.15 M cacodylate buffer at 4 °C for 8–24 h before staining.

For hemisphere samples, brains were cut with a razor blade (Wilkinson) along the midline. The hemispheres were stored in 0.15 M cacodylate buffer at 4 °C for 24 h before staining.

### Human sample

The human brain tissue sample (human individual H6, same as in ref. ^24^) was collected during a neurosurgical procedure that was indicated for medical reasons and independently from this research project at the Department of Neurosurgery at the Klinikum rechts der Isar of the Technical University of Munich. The sample was obtained from access tissue (presumably healthy brain parenchyma that had to be removed as part of the procedure and would have been discarded otherwise) before removal of the respective target lesion, as approved by the Ethics Committee of the Technical University of Munich School of Medicine (Ethikvotum 184/16S and 273/21 S-EB). All patients had given their written informed consent.

The human ‘H6’ sample was obtained from the inferior frontal gyrus from a 69-year old female patient during surgical removal of a frontal mass lesion (final diagnosis, glioblastoma multiforme), the same patient as in ref. ^24^. Following surgical removal, tissue was directly collected in fix solution kept at 4 °C. The tissue was immediately sliced into 2-mm-thin slices in cold fixative using a vibratome. Slices were kept at 4 °C overnight. Samples sized 2 × 3 mm^2^ were then cut out from the slices with razor blades.

The 2 × 3 × 2-mm^3^ samples were stained based on the 2-mm protocol (Extended Data Fig. 7) with minor modifications (Supplementary Table 1). In brief, after four rinses for 30 min with 0.15 M sodium cacodylate buffer (CaC) (4 °C), the following steps were applied sequentially: 22 h, 2% OsO_4_ in 0.15 M CaC (pH 7.4) at room temperature (RT); four rinses for 30 min with 0.15 M CaC at RT; 22 h, 2.5% FeCN in 0.15 M CaC (pH 7.4) at RT; 6 h, 2% OsO_4_ in 0.15 M CaC (pH 7.4) at RT; 30 min, 0.15 M CaC; three rinses for 20 min with water at RT; 18 h, 2% Pg in water at RT; three rinses for 20 min with water; 6 h, 2% OsO_4_ in water; three rinses for 20 min with water; 14 h, 4% uranyl acetate (UA) at 4 °C (afterward, switch temperature to 50 °C for 2 h); three times for 20 min with water. Afterward, the samples went through graded ethanol dehydration steps: 30 min, 20% ethanol at 4 °C; 30 min, 40% ethanol at 4 °C; 30 min, 60% ethanol at 4 °C; 30 min, 80% ethanol at 4 °C; 45 min, 100% ethanol at RT. For the resin infiltration, after three rinses for 45 min with pure acetone, the samples went through graded Epon resin steps (for 10 ml resin: 5.9 g Epon medium, 2.25 g DDSA, 3.7 g MNA, 205 µl DMP) in acetone, all at 4 °C: 4 h, 12.5%; 13 h, 25% (overnight); 4 h, 37.5%; 4 h, 50%; 19 h, 62.5% (overnight); 8 h, 75%; 19 h, 87.5% (overnight); 8 h, 95%; 19 h, 95% (first overnight for 95%); 8 h, 95%; 19 h, 95% (second overnight for 95%); 8 h, 95%; 19 h, 95% (third overnight for 95%); 8 h, 100%; 19 h, 100% (first overnight for 100%); 8 h, 100%; 19 h, 100% (second overnight for 100%); afterward, the samples were embedded in freshly prepared Epon resin and cured at 60 °C for 3 d.

### Staining experiments

All staining and resin-infiltration steps for 2-mm samples were carried out in 2-ml Eppendorf tubes and, for hemisphere or whole-brain samples, in 50-ml glass tubes at RT (~20–23 °C) unless specified otherwise. For steps involving photosensitive chemicals (FeCN, TCH, Pg, UA or lead aspartate (Ld)), the tubes were covered with aluminum foil.

All chemicals used in the staining pipeline are listed in Supplementary Table 2; all experiments reported in the main and supplementary figures are detailed in Supplementary Table 3.

For simplicity, the following terms were used to refer to the recurring staining steps in different experiments: CaC, sodium cacodylate buffer rinse, 0.15 M, pH 7.4; water, water (Milli-Q) rinse; first Os, the first OsO_4_ incubation step, 2% OsO_4_ in 0.15 M CaC, pH 7.4; FeCN, the FeCN incubation step, 2.5% in 0.15 M CaC, pH 7.4; second Os, the OsO_4_ incubation step after FeCN, 2% OsO_4_ in 0.15 M CaC, pH 7.4; Pg, the Pg incubation step, 4% in water; TCH, the TCH incubation step, 1% in water; third Os, the OsO_4_ incubation step after Pg, 2% in water; UA, the UA incubation step, UA (2% or 4%) dissolved in water; Ld, the Ld incubation step, 0.66% lead nitrate in 0.03 M aspartic acid, pH 5.0.

### Staining of 2-mm samples with the 1-mm protocol

The 1-mm staining protocol (ref. ^6^ with addition of the second Os step) was applied to 2-mm samples directly. The samples were stained with the following steps: first Os, 1.5 h; FeCN, 1.5 h; second Os, 1 h; CaC, 0.5 h; water, 0.5 h; TCH, 1.5 h; water, 0.5 h (two times); third Os, 1.5 h; water, 0.5 h (two times); 2% UA (17 h at 4 °C, 50 h at 50 °C); water, 0.5 h (two times); Ld, 2 h at 50 °C; water, 0.5 h (two times). Afterward, the samples were incubated in a graded ethanol series from 50% (4 °C) to 75% (4 °C) to 100% (each step, 45 min). Next, the samples were incubated in pure acetone three times, each time for 45 min. Afterward, they were incubated in 50% Spurr’s resin (Sigma-Aldrich, at a ratio of 0.95 g ERL-4221, 5.9 g DER 736, 0.1 g NSA, 113 µl DMAE) in acetone for 6 h. Next, the samples were left overnight with the cap of the Eppendorf tubes open to allow evaporation of acetone. Afterward, the samples were transferred into pure Spurr’s resin for 6 h before embedding and curing at 70 °C for 1–3 d. After resin curing, the samples were first imaged by μCT for staining homogeneity. Next, they were trimmed to expose the sample center; the sample surface was smoothed and imaged in high-vacuum SEM and energy-dispersive X-ray spectroscopy (EDS).

### Step-by-step μCT diagnosis of the main staining steps of the 1-mm protocol on 2-mm samples

The 1-mm staining protocol (ref. ^6^ with the addition of the second Os step) was applied to a batch of 2-mm samples as described above: first Os, 1.5 h; FeCN, 1.5 h; second Os, 1 h; CaC, 0.5 h; water, 0.5 h; TCH, 1.5 h; water, 0.5 h (two times); third Os, 1.5 h; water, 0.5 h (two times); 2% UA (17 h at 4 °C, 50 h at 50 °C); water, 0.5 h (two times); Ld, 2 h at 50 °C; water, 0.5 h (two times). After each main step (first Os, FeCN, second Os, third Os, UA), two samples were taken out from the staining pipeline and rinsed with either CaC or water, depending on the solvent condition of the corresponding staining step (rinsing solution was changed every 8 or 17 h). When the rinsing was complete for all samples from different conditions, samples were embedded in 2% agarose in water in the same Eppendorf tube at different tube depths and stored at 4 °C for the agarose to cure. Next, the tube containing all the samples was imaged by μCT to investigate staining gradients.

### Extending the FeCN incubation time for 2-mm samples

The 2-mm samples were stained as follows: first Os, 3 h; FeCN. During the FeCN incubation, two samples were taken out at each of the following time points: 1.5 h, 3 h, 7 h and 17 h. Samples were then rinsed with CaC for 1 h and embedded in 2% agarose as described above. After agarose curing, they were imaged by μCT to investigate staining gradients possibly related to FeCN incubation.

### Interaction between OsO_4_ incubation duration and FeCN incubation

The 2-mm samples were stained using the following two conditions: (1) first Os, 3 h; FeCN, 17 h or (2) first Os, 24 h; FeCN, 17 h. Next, they were rinsed sequentially with CaC (for 0.5 h) and water (two times, each time for 0.5 h); they were then dehydrated and embedded according to the 2-mm Spurr’s resin protocol. After resin embedding, they were trimmed to expose the center, their surface was smoothed, and they were imaged by low-vacuum SEM to investigate membrane contrast.

### Extending the TCH incubation for 2-mm samples

Three groups of 2-mm samples were stained; each batch was incubated with TCH for a different incubation length, using the following protocol: first Os, 3 h; FeCN, 17 h; second Os, 3 h; CaC, 0.5 h; water, 0.5 h (two times); TCH for 1.5 h or 3 h or 5 h; water, 0.5 h (two times); third Os, 3 h. Afterward, they were rinsed and embedded in 2% agarose for imaging by μCT.

### Replacing TCH with pyrogallol

Three groups of 1-mm samples were stained; each group differed in the TCH-related step, using the following protocol: first Os, 1.5 h; FeCN, 1.5 h; second Os, 1 h; CaC, 0.5 h; water, 0.5 h; TCH, 1.5 h or Pg, 1.5 h or water, 1.5 h; water, 0.5 h (two times); third Os, 1.5 h; water, 0.5 h (two times); 2% UA (17 h at 4 °C, 50 h at 50 °C); water, 0.5 h (two times); Ld, 2 h at 50 °C; water, 0.5 h (two times). Afterward, they were dehydrated and embedded in resin according to ref. ^6^. After resin curing, the samples were trimmed to expose the center, their surface was smoothed, and they were imaged by high-vacuum SEM and EDS. For EDS measurement, we selected point measurements in the neuropile of the sample center.

### Comparison of pyrogallol incubation in water versus CaC

Two groups of 2-mm samples (the groups differ in Pg incubation and the water steps around Pg) were stained with the following sequential incubation steps: first Os, 24 h; FeCN, 17 h; second Os, 3 h; CaC, 0.5 h; water, 0.5 h (twice); Pg in water, 17 h; water, 0.5 h (twice) or CaC, 0.5 h (twice); Pg in CaC, 17 h; CaC, 0.5 h (twice); third Os, 6 h; water, 0.5 h (twice); 4% UA (17 h at 4 °C, 2 h at 50 °C); water, 0.5 h (twice). Samples were then dehydrated and embedded in Spurr’s resin as described above for 2-mm samples. After resin curing, they were trimmed to expose the center, their surface was smoothed, and they were imaged by high-vacuum SEM.

### Long-duration OsO_4_ incubation for 2-mm samples

The 2-mm samples were stained with 2% OsO_4_ in CaC for either 3 d or 6 d. After rinsing with 0.15 M CaC for 0.5 h and two times with water (each time for 0.5 h), the samples were dehydrated and embedded according to the 2-mm Spurr’s resin protocol. Next, they were trimmed to expose the center, their surface was smoothed, and they were imaged by low-vacuum SEM for determining ultrastructural preservation.

### Extended pyrogallol and the third OsO_4_ incubation for 2-mm samples

Three groups of 2-mm samples (groups differ in the incubation duration of Pg and third Os steps) were stained using the following protocol: first Os, 3 h; FeCN, 17 h; second Os, 3 h; CaC, 0.5 h; water, 0.5 h (twice); Pg in water (for 6 or 17 h); water, 0.5 h (twice); third Os (for 3 or 6 h); water, 0.5 h (twice); 2% UA (17 h at 4 °C, 2 h at 50 °C); water, 0.5 h (twice). The combinations of Pg and third Os incubation times were as follows: (1) Pg, 6 h; third Os, 3 h; (2) Pg, 17 h; third Os, 3 h; (3) Pg, 17 h; third Os, 6 h. After staining, the samples were dehydrated and embedded in Spurr’s resin as described above for 2-mm samples. After resin curing, they were trimmed to expose the center, their surface was smoothed, and they were imaged by high-vacuum SEM.

### UA and Ld steps for 2-mm samples

Four groups of 2-mm samples (groups differ in incubation of UA and Ld steps) were stained using the following protocol: first Os, 3 h; FeCN, 17 h; second Os, 3 h; CaC, 0.5 h; water, 0.5 h (twice); Pg in water (17 h); water, 0.5 h (twice); third Os, 6 h; water, 0.5 h (twice); 2% or 4% UA (17 h at 4 °C, 2 h at 50 °C); water, 0.5 h (twice); Ld, 50 °C, 4 h or 24 h; water, 0.5 h (twice). The combinations of UA and Ld incubations were as follows: (1) 2% UA; no Ld; (2) 2% UA; Ld, 4 h; (3) 4% UA; no Ld; (4) 4% UA; Ld, 24 h. After staining, the samples were dehydrated and embedded in Spurr’s resin as described above for 2-mm samples. After resin curing, samples were imaged by μCT and then trimmed to expose the center; their surface was smoothed and imaged by high-vacuum SEM.

### Temperature of the OsO_4_ incubation step

Two groups of 2-mm samples were stained according to the following steps: (1) Os (4 °C, 7 d) or (2) Os (4 °C, 7 d; RT, 1 d). After staining, the samples were dehydrated and embedded in Spurr’s resin as described above for 2-mm samples. After resin curing, they were trimmed to expose the center, their surface was smoothed, and they were then imaged by low-vacuum SEM.

### OsO_4_ incubation at 4 °C followed by FeCN incubation

Four groups of samples were stained according to the following steps: (1) Os (4 °C, 6 d; RT, 1 d), FeCN (RT, 1 d); (2) Os (4 °C, 6 d; RT, 1 d), FeCN (4 °C, 1 d); (3) Os (4 °C, 7 d), FeCN (RT, 1 d); (4) Os (4 °C, 7 d), FeCN (4 °C, 1 d). After staining, the samples were dehydrated and embedded in Spurr’s resin as described above for 2-mm samples. After resin curing, they were trimmed to expose the center, their surface was smoothed, and they were imaged by low-vacuum SEM.

### Diffusion of OsO_4_ at 4 °C in hemisphere samples

A hemisphere was incubated in OsO_4_ at 4 °C. At different time points (17 h, 24 h, 40 h), it was taken out of the fridge to perform a fast μCT scan (usually about 15–20 min in total) and then returned to the fridge at 4 °C. µCT images were analyzed using Zeiss TXM3DViewer software, in which the depth of OsO_4_ diffusion was measured in sagittal reslices.

### Effect of CaC steps on the staining gradient in hemispheres

Two groups of hemispheres were stained with the following two conditions: (1) Os, 48 h; FeCN, 48 h or (2) Os, 48 h; CaC, 48 h; FeCN, 48 h. After staining, the hemispheres were briefly rinsed with CaC and cut into coronal sections of about 2 mm in thickness with a razor blade. Afterward, the coronal sections were dehydrated and embedded in Spurr’s resin as described above for 2-mm samples. After resin curing, they were imaged by μCT and then trimmed flat to expose the top surface and smoothed and imaged by low-vacuum SEM.

### Effect of CaC steps on the velocity of FeCN diffusion

Two groups of 2-mm samples were stained with the following two conditions: (1) Os, 24 h; FeCN, 1.5 h or (2) Os, 24 h; CaC, 24 h; FeCN, 1.5 h. After staining, the samples were dehydrated and embedded in Spurr’s resin as described above for 2-mm samples. After resin curing, they were imaged by μCT and then trimmed flat to expose the top surface and smoothed and imaged by low-vacuum SEM and EDS. For EDS measurement, we selected point measurements in the neuropile of the sample center.

### Interaction of pyrogallol incubation with the Os–FeCN gradient (H3)

A hemisphere sample (H3) was stained according to the following steps: first Os (4 °C, 96 h; RT, 24 h), CaC (4 °C, 48 h); FeCN (4 °C, 48 h); second Os (72 h); CaC (24 h); water (29 h); Pg (24 h); water (48 h); third Os (48 h); 4% UA (4 °C, 48 h; 50 °C, 5 h); water (42 h). For all CaC and water steps, the corresponding solutions were changed every 4 h or overnight (that is, once in the morning, noon and afternoon). During staining, the hemispheres were imaged by μCT at the main staining steps (after FeCN, after second Os, after Pg). After staining, the hemisphere was cut into coronal sections about 2 mm thick with a razor blade. Afterward, the coronal sections were dehydrated and embedded in Spurr’s resin as described above for 2-mm samples. After resin curing, they were trimmed flat to expose the top surface, smoothed and imaged by high-vacuum SEM.

### Interaction of pyrogallol incubation with the Os–FeCN gradient and CaC (H1 and H2)

Two hemisphere samples (H1 and H2) were stained according to the following steps: first Os (4 °C, 63 h; RT, 24 h); CaC (4 °C, 24 h); FeCN (4 °C, 48 h); CaC (24 h); second Os (32 h); CaC (17 h); water (9 h); Pg (48 h); water (40 h); third Os (48 h); 4% UA (4 °C, 48 h; 50 °C, 5 h); water (48 h). For all CaC and water steps, the corresponding solutions were changed every 4 h or overnight (that is, once in the morning, noon and afternoon). During staining, the hemispheres were imaged by μCT at the main staining steps (after FeCN, after second Os, after Pg). After staining, the hemisphere was cut into coronal sections about 2 mm thick with a razor blade. Afterward, the coronal sections were dehydrated and embedded in Spurr’s resin as described above for 2-mm samples. After resin curing, they were trimmed flat to expose the top surface, smoothed and imaged by high-vacuum SEM.

### Effect of CaC incubation at room temperature for 2 d on the Os–FeCN gradient (H4–H6)

Three hemisphere samples (H4–H6) were stained according to the following steps: first Os (4 °C, 72 h; RT, 24 h); CaC (48 h); FeCN (4 °C, 48 h); CaC (24 h); second Os (48 h); CaC (24 h); water (24 h); Pg (24 h); water (24 h); third Os (24 h); 4% UA (4 °C, 48 h; 50 °C, 5 h); water (48 h). For all CaC and water steps, the corresponding solutions were changed every 4 h or overnight (that is, once in the morning, noon and afternoon). During staining, the hemispheres were imaged by μCT at the main staining steps (after FeCN, after second Os, after Pg). After staining, H5 was cut into coronal sections about 2 mm thick with a razor blade. Afterward, the coronal sections were dehydrated and embedded in Spurr’s resin as described above for 2-mm samples. H4 and H6 were embedded in Spurr’s resin. After resin curing, H5 was trimmed flat to expose the top surface, smoothed and imaged by high-vacuum SEM. H4 and H6 were trimmed to expose the center, and the surface was smoothed and imaged by high-vacuum SEM.

### Effect of CaC incubation at 4 °C for 4 d on the Os–FeCN gradient (H13)

A hemisphere sample (H13) was stained according to the following steps: first Os (4 °C, 68 h); CaC (4 °C, 96 h); FeCN (4 °C, 72 h); CaC (48 h); second Os (48 h); CaC (24 h); water (24 h); Pg (24 h); water (24 h); third Os (41 h); 4% UA (4 °C, 48 h; 50 °C, 12 h); water (24 h). For all CaC and water steps, the corresponding solutions were changed every 4 h or overnight (that is, once in the morning, noon and afternoon). During staining, the hemispheres were imaged by μCT at the main staining steps (after the second Os step and after Pg). After staining, the hemisphere was embedded according to Spurr’s resin embedding for hemispheres. After resin curing, it was trimmed to expose the center, and the surface was smoothed and imaged by high-vacuum SEM.

### Whole-brain staining (W1)

A mouse whole-brain sample (W1) was stained according to the following steps: first Os (4 °C, 96 h); CaC (4 °C, 168 h); FeCN (4 °C, 72 h); CaC (4 °C, 48 h; RT, 48 h); second Os (48 h); CaC (72 h); water (48 h); Pg (24 h); water (48 h); third Os (96 h); 4% UA (4 °C, 48 h; 50 °C, 5.5 h); water (24 h). For all CaC and water steps, the corresponding solutions were changed every 4 h or overnight (that is, once in the morning, noon and afternoon). During staining, the brain was imaged by μCT at the main staining steps (during the first Os step, after the second Os step, after Pg). After staining, the brain was cut into coronal sections ~2 mm thick. Next, the sections were embedded according to Spurr’s resin embedding for 2-mm samples. After resin curing, the sections were trimmed flat to expose the surface, smoothed and checked by high-vacuum SEM.

### Effect of the second Os step on sample stability in water

Two groups of 2-mm samples (the groups differed in whether they were exposed to a second Os step) were incubated under the following conditions: (1) first Os, 24 h; CaC, 24 h; FeCN, 24 h; second Os, 3 h; CaC, 0.5 h; water (two times, 0.5 h); water or (2) first Os, 24 h; CaC, 24 h; FeCN, 24 h; CaC, 0.5 h; water (two times, 0.5 h); water. Afterward, the samples were kept in water and imaged with the light microscope at different time points (from 2 h to 100 h) to investigate macroscopic sample integrity.

### Pyrogallol–osmium(VI) interaction

For Extended Data Fig. 6, mouse hemisphere samples were stained according to one of the following options: (1) Os, 4 °C, 72 h; CaC, 4 °C, 96 h; FeCN, 4 °C, 72 h; CaC, 48 h; Os, 48 h; CaC, 24 h; CaC, 24 h; Pg, 24 h; (2) Os, 4 °C, 72 h; CaC, 4 °C, 96 h; FeCN, 4 °C, 72 h; CaC, 48 h; CaC, 48 h; CaC, 24 h; CaC, 24 h; Pg, 24 h; (3) Os, 4 °C, 72 h; CaC, 4 °C, 96 h; FeCN, 4 °C, 72 h; CaC, 48 h; Os, 48 h; KCl, 24 h; KCl, 24 h; Pg, 24 h; (4) Os, 4 °C, 72 h; CaC, 4 °C, 96 h; FeCN, 4 °C, 72 h; CaC, 48 h; Os, 48 h; KCl, 24 h, pH 7; KCl, 24 h, pH 7; KCl, pH 1; KCl, 24 h, pH 7; Pg, 24 h. After Pg, the samples were scanned by μCT with the parameters described in the section X-ray microtomography volumetric imaging of samples without resin embedding.

Afterward, the hemisphere sample from condition (2) was cut into ~2-mm coronal chunks for further processing. The coronal sections were rinsed with CaC for 24 h and then stained with either Os in water or Os in CaC for 24 h. Afterward, they were embedded according to the 2-mm Spurr’s resin-embedding protocol. After the resins were cured, the samples were cut into halves and checked with low-vacuum SEM for membrane contrast.

### Dehydration of 2-mm samples

After the last water rinsing step in the staining protocol, 2-mm samples were exposed to graded dehydration series in ethanol (50%, 4 °C; 75%, 4 °C; and 100%, RT; each step for 45 min) and then three rounds of acetone incubation (each 45 min).

### Dehydration of hemispheres

After the last water rinsing step in the staining protocol, hemisphere samples were exposed to graded dehydration series in ethanol (25%, 4 °C; 50%, 4 °C; 75%, 4 °C; and 100%, RT; each step for 8 h or overnight) and then three rounds of acetone incubation (each time for 8 h or overnight).

### Dehydration of whole-brain samples

After the last water rinsing step in the staining protocol, whole-brain samples (for example, W3) for subsequent full-volume embedding were exposed to graded dehydration series in ethanol (25% at 4 °C for 12 h, 50%, 75% and 100%; each step at 4 °C and for 24 h) and then three rounds of acetone incubation (each time for 24 h).

### Infiltration of Spurr’s resin and embedding of 2-mm samples

After dehydration, a graded incubation of Spurr’s resin (0.95 g ERL-4221, 5.9 g DER 736, 0.1 g NSA, 113 µl DMAE) was applied at 25%, 50% and 75% in acetone (each step for 8 h or overnight). Afterward, the samples were incubated for four rounds in 100% Spurr’s resin (each time for 8 h or overnight). Finally, they were embedded in freshly prepared Spurr’s resin and cured at 70 °C for 1–3 d. All steps were performed at 4 °C. For each resin exchange, the tubes were taken out from the fridge 20–30 min before to be warmed to RT.

### Infiltration of Spurr’s resin and embedding of hemisphere samples

After dehydration, a graded incubation of Spurr’s resin (0.95 g ERL-4221, 5.9 g DER 736, 0.1 g NSA, 113 µl DMAE) was applied at 25%, 50% and 75% in acetone (each step for 24 h). Afterward, the samples were incubated for 2 d in 90% resin, for 3 d in 95% resin and for 3 d in 100% resin (changing every 8 h or overnight for 90%, 95% and 100% resin steps). Finally, they were embedded in freshly prepared Spurr’s resin and cured at 70 °C for 1–3 d. All resin steps were performed at 4 °C. For each resin exchange, the tubes were taken out from the fridge 20–30 min before to be warmed to RT.

### Infiltration of Spurr’s resin and full-volume embedding of whole-brain samples

After dehydration, a graded incubation of Spurr’s resin (0.95 g ERL-4221, 5.9 g DER 736, 0.1 g NSA, 113 µl DMAE) was applied at 25%, 50% and 75% in acetone (25% and 50% for 2 d and 75% for 3 d). Afterward, whole-brain samples (for example, W3) were incubated for 2 d in 90% resin, for 4 d in 95% resin and for 6 d in 100% resin. The solutions with 95% and 100% resin were changed every 2 d. Finally, they were embedded in freshly prepared Spurr’s resin and cured at 70 °C for 3 d. All resin steps were performed at 4 °C. For each resin exchange, the tubes were taken out from the fridge 20–30 min before to be warmed to RT.

### Measurement of fluidity of Epon resin

Ten milliliters of Epon resin (Sigma-Aldrich, with a ratio of 5.9 g Epon medium, 2.25 g DDSA, 3.7 g MNA and 205 µl DMP) was prepared and stored in 15-ml Falcon tubes for different conditions: pure resin at RT (*n* = 2, green curves in Extended Data Fig. 1k), pure resin at 4 °C (*n* = 3, blue curves in Extended Data Fig. 1k) and 95% resin with 5% acetone at 4 °C (*n* = 4, red curves in Extended Data Fig. 1k). At different time points, videos were acquired of the resin moving in the tube after being turned upside down. The distance of movement of the resin in the tube was measured using the ticks (ml) printed on the Falcon tubes. The speed of resin movement was used as a measurement of the resin’s fluidity.

### Infiltration with Epon resin and embedding of 2-mm samples

After dehydration, samples were incubated in pure acetone three times, each time for 45 min. Next, a graded incubation of Epon resin (5.9 g Epon medium, 2.25 g DDSA, 3.7 g MNA, 205 µl DMP) was applied at 12.5%, 25%, 37.5%, 50%, 62.5%,75% and 87.5% in acetone (each step for 4 h or overnight). Afterward, the samples were incubated for four to six rounds in 95% Epon resin (each time for 8 h or overnight) and then for four rounds in 100% Epon resin (each time for 8 h or overnight) before embedding for curing at 60 °C for 1–3 d. All resin steps were carried out at 4 °C. For each resin exchange, the tubes were taken out from the fridge 20–30 min before for warming to RT.

### Light microscopic imaging of the sample surface to assess resin gradient

Resin-embedded samples (2-mm hemispheres) were trimmed to expose the center, and the surface was smoothed with a diamond knife. Light microscopic images of the sample surface were acquired with a slight tilt of the imaging plane, such that the otherwise black sample surface appeared silver colored.

### X-ray microtomography volumetric imaging of samples without resin embedding

To allow imaging by µCT without the need for resin embedding, 2-mm samples were embedded instead in 2% agarose (Sigma-Aldrich) in 0.15 M CaC or in water (depending on the last staining step that the sample was exposed to) in 2-ml Eppendorf tubes. They were imaged by μCT (Zeiss Xradia 520 Versa) using a voltage of 80 kV at a voxel size of 3–6 µm, using Zeiss Scout-and-Scan software (version 16.1.14271.44713). For µCT imaging of hemispheres, the samples were kept in the 50-ml glass tubes. Next, the glass tubes were put into a 140-ml syringe to be kept stable during µCT imaging (using a voxel size of 10–60 µm). µCT datasets were visualized with Zeiss TXM3DViewer software (version 1.2.10).

### Low-vacuum SEM imaging of incompletely stained samples

To investigate samples at intermediate staining steps without complete staining (and therefore often reduced signal and conductivity), they were embedded in resin. Next, they were trimmed with a diamond head trimmer (Leica EM TRIM2) to expose the center to the block faces of samples; the block face was smoothed with a diamond knife ultramicrotome (Leica EM UC7) and then imaged in a scanning electron microscope with a field-emission cathode and low-vacuum mode (Quanta FEG 450, FEI). The chamber pressure was set to 30 Pa. For the incident electron beam, a spot size of 3.5 at aperture 5 and an acceleration energy of 5 keV (measured beam current, 67 pA) were used for imaging at a pixel dwell time of 8–20 μs and a pixel size of 11.24 nm^2^ in plane (corresponding electron dose, 26–66 e^−^ nm^−2^) or 5.62 nm^2^ in plane (corresponding electron dose, 105–264 e^−^ nm^−2^), at a working distance of about 5 mm using the back scattered electron CBS detector.

### High-vacuum SEM imaging of completely stained samples

For those samples that were fully stained and embedded in resin (and therefore were expected to show sufficient conductivity), trimming and smoothing were similar to those of the previous section, but SEM imaging was performed in high-vacuum mode (5 × 10^−4^ Pa, Quanta FEG 420 and Quanta FEG 200). For the incident electron beam, a spot size of 3.2–3.5 and an acceleration energy of 2.8 keV at aperture 4–5 (beam current, 72–160 pA) were used. The pixel size was either 11.24 nm (Quanta FEG 420; aperture 4; spot size, 3.5; pixel dwell time, 4–6 µs; 31–47 e^−^ nm^−2^) (Quanta FEG 200; aperture 5; spot size, 3.2; dwell time, 8 µs; 28 e^−^ nm^−2^) or 5.62 nm (Quanta FEG 200; aperture 5; spot size, 3.2; dwell time, 8 µs; 113 e^−^ nm^−2^).

### Energy-dispersive X-ray spectroscopy (EDS) analysis

Resin-embedded samples were trimmed to expose the center as a block face (Leica EM TRIM2), smoothed (Leica EM UC7) and coated with a 10-nm gold layer (Leica EM ACE600); afterward, they were imaged in a scanning electron microscope (Amray 1830) equipped with an Si(Li) EDS detector. An incident electron beam with an energy of 18 keV was used at working distance of 15–20 mm and a takeoff angle of 20.4°. The spectrum-collection time was 20–90 s.

### UV–visible spectrum acquisition of osmium(VI) solution

We prepared 1% potassium osmate(VI) in 0.15 M sodium cacodylate buffer by adding 0.05 g potassium osmate(VI) powder (Sigma-Aldrich) into 5 ml cacodylate buffer. The solution was diluted 100 times and put into a glass cuvette to avoid spectrum signal clipping. The measurement was performed using the wavelength range 190–1,400 nm on a UV–visible spectrometer (Jasco, V-670). Measurements on the same solution were made at time points 2 min, 2 h and 24 h.

### Raman spectrum measurement of osmium(VI) and FeCN solutions

We measured the following chemicals without dilution in a custom-built Raman spectrometer (Chemistry Department of Goethe University Frankfurt) with a range of 0–4,400-cm^−1^ wavelength: (1) 0.15 M sodium cacodylate buffer; (2) 1% potassium osmate(VI) in 0.15 M cacodylate buffer; (3) 0.3% potassium osmate(VI) and 1.3% potassium ferrocyanide(II) (Sigma-Aldrich) in 0.15 M cacodylate buffer; (4) 2.5% potassium ferrocyanide(II) in 0.15 M cacodylate buffer; (5) 1.9% potassium ferricyanide(III) (Sigma-Aldrich) in 0.15 M cacodylate buffer; (6) staining solution of 2-mm samples in 2-ml Eppendorf tubes, after 24 h of 2% OsO_4_ (Serva) in 0.15 M cacodylate buffer, 1 h of 0.15 M cacodylate buffer wash and 17 h of 2.5% potassium ferrocyanide(II) in 0.15 M cacodylate buffer.

### Membrane contrast quantification

Membrane contrast was determined as the pixel-intensity differences between membrane and non-membrane regions in the EM images. For this, 500 × 500 pixel regions from the raw EM images (Fig. 1b and Extended Data Fig. 1m) were obtained and normalized for brightness (1st–99th percentiles, MATLAB 2018a, imadjust). Next, one randomly selected 500 × 500 subimage was used to train a random forest classifier (Fiji, Trainable Weka Segmentation^26^) on ImageJ (version 1.53q) to automatically identify membrane versus non-membrane voxels. Next, this classifier was applied to all subimages, and membrane contrast was calculated as the difference between median membrane and non-membrane voxel intensities. To determine differences between the measured conditions, a two-way ANOVA was performed in GraphPad Prism 9, and Tukey’s multiple comparisons across staining protocols and sampling positions were computed. Next, to determine the significance of the observed differences, we performed one-sided *t*-test for comparing the membrane contrast between the Hua protocol and our protocol (Os3 and Os24 pooled) at the core and intermediate positions, where improvements were expected.

### Three-dimensional electron microscopy imaging and image alignment

Subvolumes sized about (1.5 mm)^3^ of the hemisphere samples H6 and H13 were cut out on the Leica EM TRIM2. SBEM datasets were acquired using a custom-built SBEM microtome^27^ mounted inside the chamber of a scanning electron microscope (FEI Quanta, Thermo Fisher Scientific). The image acquisition and SBEM microtome were controlled using custom-written software^28^. An incident electron beam with an acceleration energy of 2.8 keV at aperture 4 (spot size of 3.5; beam current, 160 pA; dwell time, 2.8 µs; electron dose, 22 e^−^ nm^−2^) or aperture 6 (spot size, 3.0; beam current, 47 pA; dwell time, 12 µs; electron dose, 28 e^−^ nm^−2^) was used to image at an in-plane pixel size of (11.24 nm)^2^.

The image alignment of all EM datasets and their segmentations were carried out with routines described in ref. ^24^. The tracing of axons was carried out with webKnossos^29^. In brief, 20 axons were randomly seeded in a (3-µm)^3^ bounding box in the center of the H6 striatum dataset or from the control striatum dataset. Manual tracing was performed by three expert annotators. The occurrence of discontinuity (defined as not possible to trace non-ending nodes) was documented. The mean inter-error distance was calculated by dividing the total path length (mm) by the number of discontinuity nodes. Permutation random resampling was performed to calculate the 25th and 75th percentiles for each condition.

### Statistics and reproducibility

For the inter-error distance analysis (Fig. 2a), 20 randomly seeded axons from the dataset center were manually traced in the hemisphere and control striatum SBEM datasets, respectively. The mean inter-error distance was calculated as the total number of tracing errors divided by the total path length. Permutation (100 iterations) was performed to provide mean inter-error distance distributions for the hemisphere and control striatum datasets for the Kolmogorov–Smirnov test in MATLAB (kstest2).

For EDS analysis (Extended Data Figs. 1b,f and 4f), the elementary concentrations of Os, Fe and U (in wt %) in the neuropil region from the center of a sample were measured. Data were presented as mean ± s.d. as obtained from the EDS system. The staining experiments were replicated ≥3 times, while EDS was performed on one sample from each condition.

For the staining experiments on 2-mm and human samples, the number of replications was ≥3; for hemisphere and whole-brain staining experiments, the number of replications was ≥2 (Supplementary Table 3).

*Note added in proof: A staining and embedding protocol for 2 mm-sized human cortical samples has recently been published (Karlupia et al., 2023*, 10.1016/j.biopsych.2023.01.025*) with protocol steps partly overlapping with this protocols’ earlier version (Song et al., 2022, ref.* ^15^).

### Reporting summary

Further information on research design is available in the Nature Portfolio Reporting Summary linked to this article.

## Online content

Any methods, additional references, Nature Portfolio reporting summaries, source data, extended data, supplementary information, acknowledgements, peer review information; details of author contributions and competing interests; and statements of data and code availability are available at 10.1038/s41592-023-01866-3.

## Supplementary information

Supplementary Information Supplementary Results and Tables 1–4

Reporting Summary

Supplementary Video 1 Final sections of ATUM cutting of a fully stained and fully embedded mouse whole brain after 260 sections cut without obvious artifacts at a section thickness of 38 nm; see Fig. 1e.

## Data Availability

All 3D EM datasets are publicly available for browsing at https://webknossos.org: H6 striatum (full-stained, full-volume embedded hemisphere, Extended Data Fig. 3), https://wklink.org/7781; H13 striatum (full-stained, subvolume embedded hemisphere, Extended Data Fig. 3), https://wklink.org/7718; H13 cortex (full-stained, subvolume embedded hemisphere, Extended Data Fig. 3), https://wklink.org/7723. The µCT datasets for full-embedded hemispheres and whole brains are also publicly available for browsing at https://webknossos.org: H6, https://wklink.org/7798; H4, https://wklink.org/7732 (note that this sample has one area of mechanical damage caused by sample handling in the peri-hippocampal region); W3, https://wklink.org/7742; W1, https://wklink.org/7789 (note that this sample was imaged before resin embedding, and it has one area of mechanical damage caused by sample handling in the frontal cortex). All source data have been deposited on the Edmond platform provided by the Max Planck Digital Library^30^: 10.17617/3.RG58DU.

## Code availability

All analysis code (publicly available under the MIT license) has been deposited on the Edmond platform provided by the Max Planck Digital Library^30^: 10.17617/3.RG58DU.
