# Supplementary material for: High-contrast en bloc staining of mouse whole-brain and human brain samples for EM-based connectomics
Source: Nat Methods. 2023 May 8;20(6):836–40. doi: 10.1038/s41592-023-01866-3 (PMC10250191; doi:10.1038/s41592-023-01866-3)
Supplement: Supplementary file 1 — Supplementary Results and Tables 1–4 [file 41592_2023_1866_MOESM1_ESM.pdf]

nature methods

Brief Communication https://doi.org/10.1038/s41592-023-01866-3

High-contrast en bloc staining of mouse whole-brain and human brain samples for EM-based connectomics

In the format provided by the authors and unedited

SUPPLEMENTARY MATERIAL

Song, Feng, Helmstaedter

Supplementary Results

Detailed development of the 2mm staining protocol

When the 1 mm protocol [1] was directly applied to 2 mm sized samples, we found a two-layered gradient after OsO_4 - FeCN incubation, and an additional third gradient layer after thiocarbohydrazide (TCH)-OsO_4 incubation (Extended Data Fig. 1c). The obvious measure when observing such gradients was to extend the duration of the respective incubation steps. When extending the FeCN step from 1.5h to 12-17h, the gradient was in fact removed (Extended Data Fig. 1. d). To our surprise, however, when omitting the FeCN step entirely and only incubating in OsO_4 for 3h, membrane contrast under SEM was not reduced (and there was no gradient) (Extended Data Fig. 2a). Only when we also extended the OsO_4 incubation to 24h, the FeCN step provided enhanced membrane contrast (Extended Data Fig. 2b; for a possible explanation for this phenomenon by means of Os(vi)-CN^- coordination chemistry, see the chemical concept section and Extended Data Fig. 5l).

While the FeCN-induced gradient could thus be removed, the TCH-induced gradient could not be similarly resolved; rather, prolonged TCH incubation yielded broken samples, most likely because of gaseous products generated by the reaction of OsO_4 and TCH as reported previously[2] (Extended Data Fig. 1e).

We therefore adopted the exchange of TCH by pyrogallol (Pg) as proposed in [2]. When replacing TCH by Pg in the 1 mm protocol [1], we found sufficient sample conductivity and contrast (Extended Data Fig. 1f). We confirmed that pyrogallol incubation should be performed in H_2O instead of cacodylate buffer (CaC) [2, 3], which would otherwise cause decreased membrane contrast and resulted in a staining gradient (Extended Data Fig. 1g). In addition, we found that gradients or breakages were induced in the Pg steps unless we pre-incubated in H_2O (Extended Data Fig. 4g) or took other measures to avoid an interaction between Os(vi) and Pg (see Extended Data Fig. 6h-n). By extending the

incubation times for pyrogallol and the following OsO_4 step, the second gradient vanished (Extended Data Fig. 1h, Extended Data Fig. 7).

For the final steps in our protocol, uranyl acetate (UA) and lead aspartate (Ld), we noticed under µCT a minor gradient caused by UA (Extended Data Fig. 1i). This gradient was removed by increasing the UA concentration from 2% to 4% (Extended Data Fig. 1i). We omitted the final lead aspartate step as the low concentration of lead yielded very bad diffusibility (20 mM/L lead nitrate dissolved with 2:3 mole ratio in 30 mM/L saturated aspartate buffer [4]) (Extended Data Fig. 1i). Also under omission of the lead aspartate step, sample conductivity and staining contrast were sufficient (Fig. 1b). With this, we had obtained a high-contrast homogeneous staining protocol for 2 mm sized samples (Fig. 1b, Extended Data Fig. 1m, Extended Data Fig. 7). We further quantified the membrane contrast (Extended Data Fig. 1l, quantified as pixel intensity difference between membrane and non-membrane voxels, see also in Methods) and compared for different locations from sample stained by different Hua protocol (Fig.1b upper), our 2 mm protocol-Os3 (Extended Data Fig. 1m) and Os24 (Fig.1b lower). Membrane contrast was significantly different between conditions (two-way ANOVA and tukey's multiple comparisons) and higher for intermediate and core locations for this protocol (post-hoc one-sided t-test, n=4.75*106 voxels, p<10-4) and did not decrease for our 2 mm protocols in dependence of location within sample (Tukey's multiple comparison, p>0.4).

Development of resin protocol

We focused on Spurr's resin for samples to be cut and imaged using SBEM[5] and Epon 812 substitute (in the following referred to as Epon) for samples to be cut using ATUM[6]. We finally extended the resin (Epon) incubation time from ~1 day to 4-5 days (Extended Data Fig. 1j,k; Suppl. Table 1, which also contains data for Spurr's resin).

Hemispheres / whole brain samples

For the first OsO_4 incubation, in fact, no obvious OsO_4-based cytosolic alterations were found for samples incubated at 4°C, even when incubating in OsO_4 for 7 days (Extended Data Fig. 2e,f). With sufficiently long OsO_4 incubation at 4°C (step length was diffusion time plus about one additional day incubation for hemispheres), a step to RT was no more

necessary, and the subsequent FeCN still yielded enhanced membrane contrast. (Extended Data Fig. 2g-j). The FeCN incubation itself also induced substantial tissue damage at RT (Extended Data Fig. 2g). We therefore incubated FeCN at 4°C, as well, which resolved this problem (Extended Data Fig. 2.j).

For the FeCN step, we noticed an additional issue: especially for larger samples such as the mouse hemisphere, we observed an inverse gradient with lower µCT-imaged intensity in the periphery (Extended Data Fig. 4b), and a very slow diffusion of FeCN (Extended Data Fig. 4d). In our current understanding, this could be the result of the direct redox reaction between OsO_4 and FeCN (see Suppl. Material). When however adding washing steps with CaC between OsO_4 and FeCN incubations to avoid their direct interaction, the ensuing gradient could be removed, staining intensity in the periphery remained high (Extended Data Fig. 4c), and the FeCN diffusion was much faster (Extended Data Fig. 4e,f). We noticed that these washing steps should be at 4°C and of sufficient duration (Extended Data Fig. 4h); if they were not sufficiently long, there remained a staining gradient that would be amplified in the pyrogallol step later and may contribute to breakages (Extended Data Fig. 7, Extended Data Fig. 4g). If they were at RT, while the incubation time could be shorter (Extended Data Fig. 4h), the samples would be less stable in water with tissue damage in cerebellum (Extended Data Fig. 6f-g). Washing steps at RT also appeared to reduce the staining of postsynaptic densities (PSDs).

Chemical concepts of OsO_4-FeCN-related enhancement of membrane contrast

For enhanced membrane contrast of biological specimens in electron microscopy, potassium hexacyanoferrate(ii) (FeCN) has been combined with OsO_4 since the 1970s [7, 8]. Chemically, this was considered to involve a redox reaction between OsO_4 and FeCN[7-10], therefore the protocol has been referred to as “reduced-osmium” (rO) protocol. Two mechanisms were considered for the contrast enhancement in the rO protocol: Either more osmium compounds were deposited into membranes[8, 9, 11, 12], or osmium compounds in the cytosolic background were removed[12-14]. A more recent improved staining protocol was based on the following chemical logic[1]: The staining

process was considered as a two-step chemical reaction: First, FeCN reduces $Os^{viii}O_4$ to its lower valence form : $Os^{vi}O_2(OH)_4^{2-}$ (Os(vi)). Then, two molecules of Os(vi) dismutate into one molecule of OsO_4 and one molecule of $Os^{IV}O_2$, and OsO_2 was assumed to be able to deposit into membranes[1], thus result in higher membrane contrast.

For the protocols reported here, we developed a different chemical concept. First, we noted that several experimental results would not support the above "redox-dismutation" [1] explanation. The assumed dismutation reaction was found in either organic solvents or low pH water solution[15], but it is less likely to occur in cacodylate buffer (CaC). In support of this, we found no change in UV-Vis absorption spectrum of potassium osmate (vi) ($K_2[Os^{VI}O_2(OH)_4]$) solution in CaC after 24 h compared to freshly prepared solution (Extended Data Fig. 5d). In brain tissue, dismutation might still occur in a more complex way involving interactions with lipid membranes. However, we found that membrane contrast was not enhanced by staining samples directly with potassium osmate(vi) in CaC solution (Extended Data Fig. 5c). Furthermore, we noticed that the redox reaction between OsO_4 and FeCN as the basis for membrane contrast enhancing[1, 7-10] had little direct support. A direct test would be to determine membrane contrast with a suppressed redox reaction. When adding an additional CaC washing step to remove OsO_4 from the tissue before adding FeCN, in an attempt to suppress OsO_4-FeCN redox reactions, similar membrane contrast was observed (Extended Data Fig. 5a,b, comparing to Fig.1b and Extended Data Fig. 1m); indicating that the OsO_4-FeCN redox reaction may not be a prerequisite for enhanced membrane contrast.

For developing an alternative explanation of membrane contrast enhancement, we were guided by the observation that the duration of the OsO_4 incubation step was critical for the effect of FeCN: when the OsO_4 incubation duration was short (but sufficient to stain a 2 mm-sized sample homogeneously, e.g. 3 hours), addition of FeCN actually did not enhance membrane contrast (Extended Data Fig. 2a); only when prolonging the OsO_4 incubation to 24 h, the subsequent FeCN step was effective in membrane contrast enhancement (Extended Data Fig. 2b). Which processes could have occurred during the prolonged OsO_4 incubation? After short incubation, both the membranes and background appeared stained (Extended Data Fig. 2a), implying that osmium compounds were

deposited/attached at both targets. After prolonged incubation, however, the cytosolic background (which may mainly be constituted by proteins), may have been altered by OsO_4 such that FeCN can react to yield products that can be washed out, reducing background staining (Extended Data Fig. 2b; consistent with insights in earlier studies[12-14]). One possible process/reaction that may have happened between OsO_4 and background is protein over-oxidization[16], which might induce 3D -conformational changes of the proteins (e.g. de-gelation[17]). As a result, more protein-bound osmium (of valence vi[16]) could be exposed to the aqueous phase, enabling the interaction with FeCN in the aqueous solution. This notion of conformational instability of proteins as a prerequisite for FeCN effects was supported by an experiment in which we added $CaCl_2$ as a protein crosslinking cation[16], and indeed found this to inhibit the membrane contrast enhancement effect of FeCN after prolonged OsO_4 incubation (Extended Data Fig. 5g). In addition, when incubating at 4 °C, protein conformation would be expected more stable, and chemical reactions slowed down. In fact, low-temperature incubation could also inhibit the effect of FeCN on membrane contrast after prolonged OsO_4 incubation (Extended Data Fig. 5h).

But is the addition of FeCN a necessity after prolonged OsO_4 exposure for enhancement of membrane contrast, or could sufficiently long OsO_4 incubation alone provide sufficient membrane contrast? We stained samples with further extended OsO_4 incubation (from 24 h to 3-6 days) and investigated membrane contrast afterwards in low-vacuum SEM. After 6 days of OsO_4 incubation, enhanced membrane contrast was in fact achieved (Extended Data Fig. 2c,d). However, extensive background extractions also occurred (Extended Data Fig. 2c,d). Together, we could interpret this as an indication that FeCN was not strictly required for enhanced membrane contrast; however it could accelerate contrast enhancement such that sufficient contrast could be achieved before the OsO_4 incubation yielded extraction of background ("over-fixation").

We still needed to form a hypothesis for the actual reactions occurring between Os-compounds and FeCN. These experiments and concepts are described in the following.

During prolonged OsO_4 incubation, we noticed a pink reaction product diffusing out from the samples (for comparison, OsO_4 was yellowish in solution) (Extended Data Fig. 5i),

the typical color of osmate(vi) in cacodylate. This further supported the previous notion that the background osmium species exposed by prolonged OsO_4 was Os(vi). During the very long OsO_4 incubation, as the proteins were further oxidized, the exposed Os(vi) might be released into aqueous phase, likely coordinated with the anion from the CaC buffer as this could stabilize Os(vi) in water (Extended Data Fig. 5d)). In this coordinated form, Os(vi) could diffuse out of the sample, reducing background staining, thus enhancing membrane contrast. This implied that this Os(vi)-removal process would be slow, as 24 h OsO_4 incubation had not yielded enhanced membrane contrast, but only 3-6 days of OsO_4 incubation (Extended Data Fig. 2c,d) - consistent with the notion from previous studies that “reduced osmium” diffusion was slow[1, 2].

How could the addition of FeCN accelerate this Os(vi) background removal process? After OsO_4 incubation we would expect Os(viii) and Os(vi) freely moving in the aqueous phase. Since we consider redox reaction or dismutation unlikely as key processes in our explanation (see above), thus excluded the possibility of reaction between Os (viii)-FeCN or Os(vi)-Os(vi); we had to considered the remaining likely chemical reaction to occur between Os(vi) and FeCN. This would be consistent with the observation that the staining solution after the FeCN step had a green-blue color, similar to the color observed in the in-vitro reaction of Os(vi) and FeCN (Extended Data Fig. 5i).

What would be a realistic reaction between Os(vi) and FeCN to occur? We explored this by measuring the Raman spectrum of the mixture solution of Os(vi) and FeCN. In the mixture, we did not find the spectral peak expected for potassium hexacyanoferratte (iii) (FeCNiii) (Extended Data Fig. 5j), indicating that Os(vi) was not able to oxidize FeCN to FeCNiii. On the other hand, Os(vi) was known to have the capacity to coordinate with ligands like OH^- [1, 18] and CN^-[18-21], and FeCN was known to be unstable such that it would dissociate to yield free CN^- spontaneously at low rate in aqueous solution[22]. Thus a possible reaction between Os(vi) and FeCN was a coordination reaction, in which Os(vi) competed for the CN^- from FeCN and formed a coordination complex.

This hypothesis was further supported by our measurement of the Raman spectroscopy: we found a shift of the vibration peak of $O{=}Os^{VI}{=}O$ in the Os(vi)-FeCN solution compared to Os(vi) alone (Extended Data Fig. 5j), indicating a ligand change in the Os(vi) molecules.

The same shifted peak was also found in the brain staining solution after the FeCN step (Extended Data Fig. 5k), indicating that FeCN in the sample staining processes could in fact react with Os(vi).

Together, a possible function of FeCN is to “carry” CN^- to diffuse through the sample and donate CN^- to exposed Os(vi) (after prolonged OsO_4 incubation). If the incubation of OsO_4 before FeCN was not sufficiently long to expose background Os(vi), FeCN incubation would only remove the free Os(vi) in solution, not the Os(vi) bound to cytosolic proteins, thus would not result in enhanced membrane contrast. If the incubation of OsO_4 was sufficiently long to expose protein-bound Os(vi), FeCN incubation would however coordinate with the exposed protein-bound Os(vi), enabling its removal from the sample, and yielding enhanced membrane contrast. The likely form of this assumed complex might be $OsO_2(OH)_2(CN)_2^{2-}$, $OsO_2(CN)_4{}^{2-}$-[20, 21].

This concept adds a new view on the long mysterious chemical nature of OsO_4-FeCN membrane contrast enhancement (Extended Data Fig. 5l): (1) OsO_4 stained both membrane and cytosolic proteins (background) during short incubation. Os(vi) would be produced in the membrane and within the extracellular matrix (ECM). While membrane-related Os(vi) would have access to the aqueous phase, protein-bound may be shielded from converting into free Os(vi). If FeCN was then applied to the sample, it would donate CN^- as coordination ligand to Os(vi); however, this would only occur for free Os(vi), since Os(vi) in the background or membranes would not yet be as accessible for FeCN. This would keep osmium in both membranes and backgrounds, resulting in no contrast enhancement or slightly reduced contrast. (2) With prolonged incubation, OsO_4 over-oxidized the background proteins, potentially causing 3D conformational change of background proteins, and thereby exposed protein-bound Os(vi) to aqueous phase; (3) FeCN would carry CN^- through the sample, and provide CN^- to the exposed protein-bound Os(vi) exposed within the cytosolic/ECM protein network to form a stable coordination compound (note this would be a compound between Os(vi) and CN^-, but not between Os(vi) and FeCN itself as proposed in 1970s[9]). The coordination compound between Os(vi) and CN^- could then easily diffuse out[11]. This would result in removal of background osmium and could thus increases local contrast.

Discussion of previous protocols in the framework of the exposure-coordination based staining concept

The possible exposure-coordination concept of membrane contrast enhancement would add a surprising view on Karnovsky[7] and de Bruijn's[8] discovery in 1970s to find FeCN as a staining enhancement agent: the reason this would have worked was that Fe^{2+} was strong enough to maintain CN^- in the coordination complex, such that CN^- would not react directly with the osmium compounds deposited within membranes (as the osmium compounds in membranes are those we would like to keep). In fact, otherwise the effect would be dramatic: the staining of the entire sample would be lost if osmicated samples were incubated in KCN which provide free CN^-[11]. FeCN, in contrast, would be more strongly coordinated and only be able to donate CN^- when encountering the exposed/free Os(vi) in the aqueous solution, forming the stable coordination product of Os(vi) and CN^- [20, 21] that would be easily washed out[11]. Our chemical concept was also consistent with the "background washing" insight from the early days[12-14].

The same incubation time dependent effect would also exists in the original rO protocol (Extended Data Fig. 5e,f). The principles summarized above can be applied similarly: even though FeCN and OsO_4 were mixed together in rO (which would yield a mixture of OsO_4, FeCN, FeCNiii and Os vi); however, FeCN-based reactions would still only be occurring once the background proteins were exposing Os(vi) for CN^- - complex formation.

To provide a possible explanation for the staining gradient and precipitation band that would appear when staining samples with rO that were larger than ~200 μm in size[1, 2], we considered the following: The very slow centripetal diffusion of Os(vi) (Extended Data Fig. 5c) from all sides of the sample could yield a density of Os(vi) at a certain depth such that CaC could no longer stabilize the high amount of Os(vi), and local dismutation would occur (thereby yielding $Os^{IV}O_2$, Extended Data Fig. 5c,d).

To evaluate the rO[7, 8], Hua[1] and Mikula[2] protocols in the framework of the exposure-coordination concept, we consider the Os(vi) coordination reactions with different ligands (hydroxide, cacodylate, cyanide and others, Extended Data Fig. 5m). The coordination bonds from different ligands would differ in their strength, influencing the stability of the

complexes. Similarly, the complexes would also differ in size as a result of different ligand sizes; this would influence the mobility of the complexes during diffusion. In water, Os(vi) would coordinate with hydroxide ions (OH^-) and form an unstable complex which would dismutate relatively easily[1, 15]. In cacodylate buffer, Os(vi) would form more stable complexes with cacodylate anions ($(CH_3)_2AsO_2^-$), very likely also by coordination bond[18], which should be stronger so that it can inhibit Os(vi) dismutation (Extended Data Fig. 5d). The difference in the size of the coordination bonds by hydroxide vs. cacodylate could also contribute: in hydroxide coordination there would be a distance of only two chemical bonds (Os-O-H)), but in cacodylate coordination there would be a distance of four chemical bonds (Os-O-As-C-H), potentially affecting final compound size and mobility, possibly explaining why the [Os(vi)-cacodylate] coordination compound diffused slowly[1, 2].

For the formamide used to facilitate rO diffusion [2, 3], Os(vi) would coordinate with the amine group in formamide[18]. The formamide coordination with Os(vi) would result in a three bond distance (Os-N-C-H), thus less than the four bond distance of cacodylate; this might give the formamide coordination compound more mobility. The coordination ligand amine group should also form relatively stronger coordination than hydroxide[18], this could help preventing precipitations as discussed in the “reduced osmium” protocol.

Remaining artifacts

Three types of artifacts were observed in the large-sample protocols: (1) Blood vessels partly detached from the surrounding neuropil (Extended Data Fig. 6b); (2) Remaining micro-breakages in subcortical neuropil (Extended Data Fig. 6d,e); (3) Damage to outer parts of the cerebellum that could break off during water incubation steps (Extended Data Fig. 6f).

For blood vessel artifacts, in order to determine in which protocol step they appeared, we imaged 2 mm samples after 24 h RT OsO_4 incubation in µCT; with this method, we avoided the influence of dehydration and resin infiltration. Blood vessels already detached after the first OsO_4 incubation step (Extended Data Fig. 6c). The result was the same for samples that was OsO_4 incubated at 4°C for 4 days (Extended Data Fig. 6c). While not a

major concern for connectomics projects aimed at synaptic circuitry, for projects in which the complete integrity of blood vessels were to be essential, we see the need for further targeted optimization of the protocol.

For micro-breakages in subcortical areas, these were rather small (≤ 500 nm in width, see in Extended Data Fig. 6d), rendering detection by µCT difficult. Since these occurred rarely and predominantly in myelin-rich areas, we expect neurite reconstruction to be largely unaffected.

For the cerebellum breakage artifacts, it was obvious from the staining process that they occured during the first H_2O incubation step (Extended Data Fig. 6g). We found this to be influenced by the temperature of CaC incubation between OsO_4 and FeCN: when the CaC incubation step was at 4°C, the cerebellum was preserved much better than at RT (Extended Data Fig. 6g).

In summary, the remaining artifacts are not expected to be problematic for connectomic analyses, but may require attention when applying our protocol to a broader set of examinations of large-scale 3D EM datasets from various tissue types, also in the pathological context.

Additional protocol variants for highly fragile samples

The protocol steps in pure H_2O create substantial osmotic stress, which can be critical for large fragile samples. While we were able to stabilize whole-brain samples around the Pg step by adding the second OsO_4 step after FeCN (Extended Data Fig. 4i), the pure H_2O incubation might still be problematic for some kinds of larger samples, for example those with very large blood vessels from the human brain. To accommodate such samples, an alternative approach would be to increase the osmolarity using chemically inactive compounds. We observed that CaC buffer would not work as an osmolarity balancer because prohibitive infiltration gradients (and breakages when gradients are too strong) would occur during the Pg step (Extended Data Fig. 4g).

One possible explanation for such gradients occurring during Pg step under CaC, not H_2O, incubation would be the following (Extended Data Fig. 4g, Extended Data Fig. 6i):

As CaC can stabilize Os(vi) (Extended Data Fig.5d), there might be an interaction between Pg and the CaC-stabilized free Os(vi) in the samples. After the FeCN step, one would not expect free Os(vi) in the tissue (Extended Data Fig.5l); the additional 2nd Os step, however, could generate residual Os(vi). To test the possible role of Os (vi)-Pg interaction in generating staining gradients, in brief, hemispheres were first stained with our final hemisphere protocol but only for the steps from 1st Os to the CaC rinse after FeCN; afterwards, alternatives were explored to control the presence of Os(vi) before the Pg step: in fact when the 2nd Os step was omitted, no staining gradient appeared in the Pg step even when there was Pg-CaC interaction (Extended Data Fig.6h,j); on the other hand, when adding KCl in H_2O, known to stabilize Os(vi) [18], a staining gradient appeared during the Pg step (Extended Data Fig. 6h,k); this could be reverted by removing Os(vi) from KCl-H_2O by adjusting the pH briefly to 1 (which triggers the dismutation of Os(vi), Extended Data Fig. 6h,i).

In summary, our experiments support the conclusion that when using Pg, special care should be taken to make sure that there is no residual free Os(vi) in the sample in order to avoid gradients (and breakages when the gradients are too large) caused by Pg-Os(vi) interactions. This understanding would, in principle, open new opportunities in choosing the incubation solution before and after Pg. For example, our pilot experiment with omitting the second Os step (In brief: 1st Os→cac→FeCN→cac→Pg→cac→Os h2o/Os cac Extended Data Fig.6h,j,m,n,) show options for samples that are especially sensitive to osmotic stress (e.g. mouse cerebellum and very large human cortical tissue samples).

Supplementary Tables

Supplementary Table 1: Detailed protocols for mouse & human 2 mm, mouse hemisphere and whole brain sample staining and resin embedding

Samples sized 2 mm were stained in 2 ml Eppendorf tubes, hemispheres / whole brains were stained in 50 ml tubes. All steps were at room temperature (RT) unless otherwise noted. From step 3 (FeCN) onwards, all tubes were covered with aluminum foil.

I. Staining

Step	Solution	2 mm	Human* (2*3*2 mm^3)	Hemisphere (H)	Whole Brain (WB)	Notes
**1a**	**OsO_4** 2% in 0.15 M CaC, pH 7.4	n/a	n/a	72 h, 4°C	96 h, 4°C	
**1b**	**OsO_4** 2% in 0.15 M CaC, pH 7.4	24 h	22 h<br><br>(2 h less in order to keep time schedule practical)	n/a	n/a	
**2**	**CaC** 0.15 M, pH 7.4	1.5-2 h, 4°C<br><br>change every 0.5 h	2 h, 4°C<br><br>change every 20 min<br><br>(rinse more frequent)	96 h, 4°C<br><br>change every 4 h/o.n.	168 h, 4°C<br><br>change every 4 h/o.n.	
**3**	**FeCN** 2.5% in 0.15 M CaC, pH 7.4	overnight (17 h) , 4°C	overnight (22 h) , 4°C<br><br>(extended)	72 h, 4°C<br><br>change every 24 h	72 h, 4°C<br><br>change every 24 h	Light sensitive. H4-6 used 48h, but 72h recommended
**4**	**CaC** 0.15 M, pH 7.4	1.5-2 h<br><br>change every 0.5 h	(omitted to fit into practical time schedule)	48 h<br><br>change every 4 h/o.n.	96 h<br><br>change every 4 h/o.n.	
**5**	**OsO_4** 2% in 0.15 M CaC, pH 7.4	3 h	6 h<br><br>(extended)	48 h	48 h	
**6**	**CaC** 0.15 M, pH 7.4	0.5-1 h<br><br>change every 0.5 h	0.5 h	24 h<br><br>change every 4 h/o.n.	72 h<br><br>change every 4 h/o.n.	

**7**	**H_2O**	1-2 h<br><br>change every 0.5 h	1 h<br><br>change every 20 min<br>(rinse more frequent)	24 h<br><br>change every 4 h/o.n.	48 h<br><br>change every 4 h/o.n.	
**8**	**Pyrogallol**<br>4% in H_2O	overnight (17 h)<br><br>change every 4h/o.n.	overnight (18 h)<br><br>change once 2 h after the initial pg to keep time schedule practical	24 h	24 h	Freshly prepared;<br>light sensitive
**9**	**H_2O**	1-2 h<br><br>change every 0.5 h	1 h<br><br>change every 20 min	24 h<br><br>change every 4 h/o.n.	48 h<br><br>change every 4 h/o.n.	
**10**	**OsO_{4,H_2O}**<br>2% in H_2O	6 h	6 h	48 h	96 h	Was 24h for H4-6, but 48h (H13) recommended
**11**	**H_2O**	1 h<br><br>change every 0.5 h	1 h<br><br>change every 0.5 h	24 h<br><br>change every 4 h/o.n.	48 h<br><br>change every 4 h/o.n.	
**12a**	**Uranyl Acetate**<br>4% in H_2O	overnight (17 h), 4°C	overnight (14 h), 4°C<br><br>(shortened to keep time schedule practical)	48 h, 4°C	48 h, 4°C	Light sensitive. UA solution was not changed for steps 12a and 12b, only the temperature was changed.
**12b**	**Uranyl Acetate**<br>4% in H_2O	2 h, 50°C	2 h, 50°C	5 h, 50°C	5 h, 50°C	

**13**	**H_2O**	1 h change every 0.5 h	1 h change every 20 min (rinse more frequent)	24 h change every 4 h/o.n.	48 h change every 4 h/o.n.	

II. Dehydration

Step	Solution	2 mm	Hemisphere/WB	Notes
1	**25% Ethanol in H_2O**	n/a	8 h/o.n., / 12 h, 4°C	
2	**50% Ethanol in H_2O**	0.5 h, 4°C	8 h/o.n. / 24 h, 4°C	
3	**75% Ethanol in H_2O**	0.5 h, 4°C	8 h/o.n./ 24 h, 4°C	
4	**Pure Ethanol**	45 min, RT	8 h/o.n., RT / 24 h, 4°C	Samples can stay in pure Ethanol or pure acetone for days (up to 1 week), if a pause is needed practically
5	**Pure Acetone**	2 h 15 min change every 45 min	32 h, RT / 3 days, RT	

III. Resin infiltration & Embedding

Spurr's resin
4.1 g ERL 4221, 0.95 g DER 736, 5.9 g NSA, 113 µl DMAE

Step	Conc. (in acetone)	2 mm	Hemisphere /WB
1	**25% resin**	8 h/o.n., 4°C	24 h / 2 days, 4°C
2	**50% resin**	8 h/o.n., 4°C	24 h / 2 days, 4°C
3	**75% resin**	8 h/o.n., 4°C	24 h / 3 days, 4°C
4	**90% resin**	n/a	2 days, 4°C, change every 8h/o.n. / 24 h
5	**95% resin**	n/a	3 days / 4 days, 4°C,

Epon resin
5.9 g Epon embedding medium, 2.25 g DDSA, 3.7 g MNA, 205 µl DMP

Step	Conc. (in acetone)	2 mm
1	**12.5% resin**	4 h/o.n., 4°C
2	**25% resin**	4 h/o.n., 4°C
3	**37.5% resin**	4 h/o.n., 4°C
4	**50% resin**	4 h/o.n., 4°C
5	**62.5% resin**	8 h/o.n., 4°C

			change every 8h/o.n. / 24 h			
**6**	**100% resin**	2 days, 4°C;<br><br>change every 8h/o.n.	3 days / 6 days, 4°C,<br><br>change every 8h/o.n. / 24 h	**6**	**75% resin**	8 h/o.n., 4°C
**7**	**embed**	70°C, 3 days		**7**	**87.5% resin**	8 h/ o.n., 4°C
For all resin steps at 4°C, the tubes were taken out of fridge for 30 min (cap closed) before change to the solution of the next step to warm up to room temperature to avoid moisture.				**8**	**95% resin**	3 days, 4°C<br><br>change every 8h/o.n.
				**9**	**100% resin**	2 days, 4°C;<br><br>change every 8h/o.n.
				**10**	**embed**	60°C, 3 days

* The dehydration and resin infiltration of the human sample can be found in the method part of the main text.

For all solution changing step, we tried to remove as much old solution as possible to make sure there was as little interaction with new solutions as possible. For samples with sizes in between the listed 2*2*2 mm^3 and hemisphere (and/or with irregular shape (e.g. 3*5*2 mm^3)), interpolation of the above steps should be applied, and 2 ml Eppendorf tubes should also be changed to larger volume tubes (e.g. 5 ml). If modifications were made based on our recommended protocol for intermediately sized volumes, we also recommend checking staining homogeneity in µCT if possible after the major staining steps (i.e., 1st OsO_4, FeCN, 2nd OsO_4, Pg, 3rd OsO_4, UA) to verify the validity of any modifications. For hemisphere samples that aimed for ATUM cutting with the Spurr's resin embedding in our protocol, elongated infiltration of 95% and 100% resin steps might be necessary to achieve the best resin quality.

Supplementary Table 2. Employed chemicals

Chemical	Name	Preparation

MilliQ water	H_2O	-
0.15 M sodium cacodylate buffer	CaC	Diluting 0.3 M CaC (Sigma-Aldrich) 1:1 with water, pH adjusted to 7.4 by 1M NaOH (Sigma-Aldrich)
2% OsO_4 in 0.15 M cacodylate buffer	Os	Diluting 4% OsO_4 (Serva) 1:1 with 0.3 M CaC (pH 7.4), no further pH adjustment
2% OsO_4 in water	Os-aqua	Diluting 4% OsO_4 (Serva) 1:1 with water
2.5% potassium ferrocyanide in 0.15 M cacodylate buffer	FeCN	Dissolving 1.25 g FeCN (Sigma-Aldrich) in 50 ml 0.15 M CaC (Sigma-Aldrich)
1% OsO_4 + 1.5% FeCN in 0.15 M CaC	rO	Diluting 2% OsO_4 (Serva) in 0.15 M CaC (Sigma-Aldrich) 1:1 with 2.5% FeCN (Sigma-Aldrich) in 0.15 M CaC (Sigma-Aldrich)
1% thiocarbohydrazide in water	TCH	Dissolving 0.5 g TCH (Sigma-Aldrich) in 50 ml water, shake for 1 h, filter before use. Light sensitive, cover with aluminum foil
3.75% pyrogallol in water	Pg	Dissolving 1.87 g Pg (Sigma-Aldrich) in water
3.75% pyrogallol in CaC	Pg-CaC	Dissolving 1.87 g Pg (Sigma-Aldrich) in 0.15 M CaC (Sigma-Aldrich)
2% uranium acetate in water	2% UA	Dissolving 1 g UA (Serva) in 50 ml water, shake for 1 h, filter before use. Light sensitive, cover with aluminum foil
4% uranium acetate in water	4% UA	Dissolving 2 g UA (Serva) in 50 ml water, shake for 1 h, filter before use. Light sensitive, cover with aluminum foil
0.66% lead aspartate	Ld	Dissolving 0.33 g lead nitrate (Sigma-Aldrich) in 50 ml 0.03 M aspartate buffer

		(pH 3.8, Serva), adjust pH to 5.0 with 1 M KOH (Sigma-Aldrich). Light sensitive, cover with aluminum foil.
0.15 M potassium chloride	KCl	Diluting 25 ml 3 M KCl (VWR) with 475 ml H_2O

Supplementary Table 3. Protocols for all staining experiments

Abbreviated protocol descriptions for all shown control and main experiments with corresponding figure panels. Protocol steps were denoted as chemical, incubation time, temperature. Incubation time in h unless specified otherwise. Temperature only noted when other than room temperature (RT). Sample screening method is indicated (µCT and/or SEM); Sample size, dehydration, infiltration and embedding steps are reported in main methods. x2: applied twice.

Exp ID	Experiment	Staining Steps (brief)	Figures
1	Staining 2 mm samples with 1 mm protocol	Os 1.5→FeCN 1.5→ Os 45min→ CaC 0.5→x2 H_2O 0.5→<br>TCH 1→ x2 H_2O 0.5→ Os-aqua1.5→ x2 H_2O 0.5→2% UA overnight 4°C, 2 h 50°C→ µCT, SEM	Fig.1b; Extended Data Fig.1a,b
2	Step-by-step diagnosis of 1 mm protocol	Os 1.5 (µCT)→FeCN 1.5 (µCT)→ Os 45min (µCT)→ CaC 0.5→x2 H_2O 0.5 →<br>TCH 1→ x2 H_2O 0.5→ Os-aqua 1.5 (µCT)→ x2 H_2O 0.5→2% UA overnight 4°C, 2 h 50 °C (µCT)	Extended Data Fig.1c
3	Extending FeCN incubation for 2 mm	Os 3→FeCN 1.5/3/7/12/17→ Os 3→ µCT	Extended Data Fig.1d
4	Os short vs. long on FeCN's effect	Os 3→ SEM<br>Os 3→ FeCN 17→SEM<br>Os 24→SEM<br>Os 24→ FeCN 17→ SEM	Extended Data Fig.2a,b
5	Extending TCH incubation for 2 mm	Os 3→FeCN 17→ Os 3→ CaC 0.5→ x2 H_2O 0.5 → TCH 1.5/3/5→ x2 H_2O 0.5→ Os-aqua 3→ µCT	Extended Data Fig.1e

6	Replacing TCH by Pg	Os 1.5→FeCN 1.5→ Os 45min→ CaC 0.5→x2 H_2O 0.5→<br>TCH/H_2O/Pyrogallol 1→ x2 H_2O 0.5→ Os acq 1.5→ x2 H_2O 0.5→ 2% UA overnight 4°C, 2 h 50°C→ SEM & EDS	Extended Data Fig.1f
7	Comparing Pg in H2O vs. CaC	Os 24→FeCN 17→ Os 3→ CaC 0.5→x2 H_2O 0.5→<br>Pg-aqua/Pg-CaC 17→ x2 H_2O 0.5→ Os-aqua 6→ x2 H_2O 0.5→ 4% UA overnight 4°C, 2 h 50°→ SEM	Extended Data Fig.1g
8	Extending pyrogallol	Os 3→FeCN 17 → Os 3 → CaC 0.5→x2 H_2O 0.5→<br>Pyrogallol 1.5/7/17→ x2 H_2O 0.5→ Os-aqua 3/6→ µCT	Extended Data Fig.1h
9	Improving UA	Os 3→FeCN 17→ Os 3→ CaC 0.5→x2 H_2O 0.5→<br>Pyrogallol 17→ x2 H_2O 0.5→ Os-aqua 6→ x2 H_2O 0.5→ 2% /4% UA overnight 4°C, 2 h 50°C→ µCT	Extended Data Fig.1i; Extended Data Fig.1m
10	Cancelling Ld	Os 3→FeCN 17→ Os 3→ CaC 0.5→x2 H_2O 0.5→<br>Pyrogallol 17→ x2 H_2O 0.5→ Os-aqua 6→ x2 H_2O 0.5→ 4% UA overnight 4°C, 2 h 50°C→ Ld 4/24 50°C→µCT	Extended Data Fig.1i
11	Os very long	Os 3 days→ SEM<br>Os 6 days→ SEM	Extended Data Fig.2c,d
12	Os 4°C vs. RT	Os 7 days 4°C→ SEM<br>Os 6 days 4°C, 1 day RT→SEM	Extended Data Fig.2e,f
13	Os 4°C then FeCN	Os 6 days 4°C, 1day RT→FeCN 1 day 4°C/RT→SEM<br>Os 7 days 4°C→FeCN 1 day 4°C/RT→SEM	Extended Data Fig.2i,j

14	Os 4°C on hemisphere	Os 4°C → uCT at 17/24/40h	Extended Data Fig. 4a
15	CaC washing removes Os-FeCN redox gradient	Os 48→FeCN48→SEM<br><br>Os48→CaC48→FeCN48→SEM	Extended Data Fig. 4b,c
16	CaC washing speed up FeCN diffusion	Os 24 → FeCN 1.5 → uCT, SEM<br><br>Os 24 → CaC 24 → FeCN 1.5 → uCT, SEM	Extended Data Fig. 4d,e
17	Pg interaction with Os-FeCN redox gradient (H3)	Os 4°C 96, RT 24→CaC 4°C 48 →FeCN 4°C 48 (μCT) → Os 72→CaC 24(μCT) → H_2O 29 (μCT) → Pg 24 (μCT) → H_2O 48 → Os 48 → 4% UA 4°C 48, 50°C 5 →H_2O 42 →SEM	Extended Data Fig. 4g
18	Pg interaction with Os-FeCN redox gradient and CaC (H1)	Os 4°C 63, RT 24 →CaC 4°C 24 → FeCN 4°C 48 → CaC 24 (μCT)→ Os 32→CaC 17 (μCT)→ H_2O 9(μCT) → Pg 48 (μCT)→ H_2O 40 → Os 48 → 4% UA 4°C 48, 50°C 5→H_2O 48→ SEM	Extended Data Fig. 4g
19	CaC RT 2d removes Os-FeCN redox gradient (H4,5,6)	Os 4°C 72, RT 24→CaC 48 → FeCN 4°C 4 → CaC 24 (μCT H5) → Os 48→CaC 24→ H_2O 24 (μCT H5) → Pg 24 (μCT H5) → H_2O 24 → Os 24 (μCT H5) → 4% UA 4°C 48, 50°C 5 →H_2O 48 →SEM	Extended Data Fig. 4h; Extended Data Fig. 3a,b,c; Extended Data Fig.6a
20	CaC washing at 4°C 4 day removes Os-FeCN redox gradient (H13)	Os 4°C 68 →CaC 4°C 96 → FeCN 4°C 72 → CaC 48 → Os 48 →CaC 24 → H_2O 24 (μCT) → Pg 24 (μCT) → H_2O 24 → Os 41 (μCT at 24/41 h) → 4% UA 4°C 48, 50°C 12 →H_2O 24 →SEM	Extended Data Fig. 4h; Extended Data Fig. 3d
21	Whole brain staining (W1,W2)	W1: Os 4°C 96 (μCT) → CaC 4°C 168→ FeCN 4°C 72 (μCT) → CaC 4°C 48, RT 48 → Os	Fig.1c;

22		48→CaC 72 (μCT) → H_2O 48 (μCT) → Pg 24 (μCT) → H_2O 48→ Os 96 (μCT) → 4% UA 4°C 48, 50°C 5.5 →H_2O 24 →SEM<br><br>W2: Os 4°C 96 → CaC RT 72, 4°C 72→ FeCN 4°C 72 → CaC 4°C 48, RT 48 → Os 48→CaC 72 → H_2O 48 (μCT) → Pg 24 → H_2O 48→ Os 96 → 4% UA 4°C 48, 50°C 5.5 →H_2O 24 →SEM	Extended Data Fig.6g
22	2nd Os stabilize samples in H_2O	Os 24 → CaC 24 → FeCN 24 → Os 3 → CaC 0.5 → x2 H_2O 0.5 → H_2O→ take LM images at different time points<br><br>Os 24 → CaC 24 → FeCn 24→ CaC 0.5 → x2 H_2O 0.5 → H_2O → take LM images at different time points	Extended Data Fig. 4i
23	Os vi staining	1% Os (vi) CaC 6→ SEM	Extended Data Fig.5c
24	Adding $CaCl_2$ in OsO_4 then FeCN	Os 24 (with $CaCl_2$)→ FeCN 17→ SEM	Extended Data Fig.5g
25	OsO_4 at 4°C then FeCN	Os 24 4°C→FeCN17→SEM	Extended Data Fig.5h
26	rO short vs. long	rO 3 → SEM<br>rO 24 → SEM	Extended Data Fig.5e,f
27	CaC wash between OsO_4 and FeCN	Os 3/24→x3 CaC 1→FeCN 17→SEM	Extended Data Fig.5a,b

28	CaC before Pg causes gradient	Os 4°C 72 →CaC 4°C 96 → FeCN 4°C 72 → CaC 48 → Os 48 →CaC 24 → CaC 24 → Pg 24 (μCT)	Extended Data Fig.6i
29	Cancelling 2nd Os to remove Os vi before Pg	Os 4°C 72 →CaC 4°C 96 → FeCN 4°C 72 → CaC 48 → CaC 48 →CaC 24 → CaC 24 → Pg 24 (μCT)<br>Afterwards, the hemisphere was cut into four ~2 mm thick coronal chunks for exp32 and exp33.	Extended Data Fig.6j
30	KCl between 2nd Os and Pg causes gradient	Os 4°C 72 →CaC 4°C 96 → FeCN 4°C 72 → CaC 48 → Os 48 →KCl 24 → KCl 24 → Pg 24 (μCT)	Extended Data Fig.6k
31	Adjusting pH of KCl to remove Os vi before Pg	Os 4°C 72 →CaC 4°C 96 → FeCN 4°C 72 → CaC 48 → Os 48 →KCl 24, pH7 → KCl 24, pH7 → KCl pH1 → KCl 24, pH7 → Pg 24 (μCT)	Extended Data Fig.6l
32	Os in h2o staining of sample from exp29	Exp29 → CaC24 → Os in h2o 24 → h2o → 2 mm dehydration & Spurr's resin embedding → μCT, SEM	Extended Data Fig.6m
33	Os in cac staining of sample from exp29	Exp29 → CaC24 → Os in cac 24 → h2o → 2 mm dehydration & Spurr's resin embedding → μCT, SEM	Extended Data Fig.6n

Supplementary Table 4. Comparison of protocols

Comparison between the main steps of rOTO (Willingham & Rutherford, 1984), Hua et al (Hua et al., 2015), Mikula et al. (Mikula & Denk 2015) and our protocol are summarized in the following table. Abbreviations are the same as previous tables.

Main steps	rOTO (Willingham et al. 1984)	Hua et al., 2015	Mikula et al. 2015	This Protocol (Song et al.)
**Membrane Contrast**	**Os + FeCN (RT)**	**Os → FeCN (RT) (RT)**	**Os+FeCN+formamide**	**Os → CaC → FeCN (4°C) (4°C) (4°C)**

			**(RT)**	
Ideas and notes about the step	(1).Os-FeCN redox is the core reaction<br><br>(2). Either more osmium deposited into membranes; or osmium got washed out from background	(1).Os-FeCN redox is the core reaction to generate Os vi<br><br>(2). Os vi dismutate to Os iv to deposited into membrane, resulting in increased contrast<br><br>Separating Os and FeCN avoids locally too high os vi concentration.	(1). Os-FeCN redox is the core reaction<br><br>(2). Formamide might act as a stabilizer for Os vi or charge neutralizer for FeCN.<br><br>Formamide added into Os-FeCN mixture helps either Os (vi) or FeCN diffusion	(1).Os-FeCN redox is not necessary<br>(2). Os vi cannot enhance membrane contrast<br>(3). Os long cause background os vi to expose to aqueous phase<br>(4). FeCN gives CN^- to background Os vi to wash it away<br><br>Adapted Hua's Os-FeCN separation; however, as Os-FeCN redox is not necessary, added CaC in between to avoid artefacts. Temperature 4°C to avoid os over-expose and further stabilize samples in later steps (except 4°C, $CaCl_2$ should also have the same effect, see in Extended Data Fig.5g,h).
**Staining Amplification**	**TCH**	**TCH**	**Pyrogallol**	**Pyrogallol**
Ideas and notes about the step	(1). TCH symmetrically link Os<br>(2). however, TCH-OsO_4 reaction generate bubbles causing breakages in large samples (>1 mm).		(1). Pg can replace TCH<br>(2). Chemistry unclear;<br>(3). Pg in H_2O better than in CaC.<br><br>However, cac was used around Pg step.	Our experiments indicate the interaction between Os (vi) and Pg cause staining gradient (Extended Data Fig. 4g, Extended Data Fig.6h-n).<br><br>Adapted Mikula's pyrogallol; however,

				we use water around Pg step;<br>Added 2nd Os to help stabilize samples in water.
**UA**	**-**	**4°C then 50°C**	**-**	**4°C then 50°C**
Ideas and notes about the step	-	Low temperature facilitate diffusion by inhibiting reaction	-	Adapted from Hua; with increased UA concentration from 2% to 4%.
Ld	-	50°C	-	Omitted due to too slow diffusion of Lead aspartate.

Supplementary References

1. Hua, Y., P. Laserstein, and M. Helmstaedter, *Large-volume en-bloc staining for electron microscopy-based connectomics.* Nature Communications, 2015. **6**: p. 1-7.
2. Mikula, S. and W. Denk, *High-resolution whole-brain staining for electron microscopic circuit reconstruction.* Nature Methods, 2015. **12**: p. 541-546.
3. Genoud, C., et al., *Fast Homogeneous En Bloc Staining of Large Tissue Samples for Volume Electron Microscopy.* Frontiers in Neuroanatomy, 2018. **12**: p. 1-8.
4. Walton, J., *Lead Aspartate, an en Bloc Contrast Stain Particularly Useful for Ultrastructural Enzymology.* The Joural of Histochemistry and Cytochemistry, 1979. **27**(10): p. 1337-1342.
5. Denk, W. and H. Horstmann, *Serial block-face scanning electron microscopy to reconstruct three-dimensional tissue nanostructure.* PLoS Biol, 2004. **2**(11): p. e329.
6. Hayworth, K.J., et al., *Automating the Collection of Ultrathin Serial Sections for Large Volume TEM Reconstructions.* Microscopy and Microanalysis, 2006. **12**(S02): p. 86-87.
7. Karnovsky, M.J., *Use of ferrocyanide-reduced osmium tetroxide in electron microscopy*, in *11th annual meeting, the American Society for Cell Biology*. 1971.
8. Bruijn, W.C.d., *Glycogen , Its Chemistry and Morphologic Appearance in the Electron Microscope I. A modified OsO4 fixative which selectively contrast glycogen.* J. Ultrastructure Research, 1973. **50**.
9. White, D.L., J.E. Mazurkiewicz, and R.J. Barrnett, *A chemical Mechanism for Tissue Staining by Osmium Tetroxide-Ferrocyanide Mixtures.* The Journal of Histochemistry and Cytochemistry, 1979: p. 1084-1091.
10. Riemersma, J.C., E.J.J. Alsbach, and W.C.D. Bruijn, *Chemical aspects of glycogen contrast-staining by potassium osmate.* Histochemical Journal, 1984. **136**: p. 123-136.
11. Bruijn, W.C.D. and P.D. Breejen, *Glycogen , its chemistry and morphological appearance in the electron microscope . II. The complex formed in the selective contrast staining of glycogen.* Histochemical Journal, 1975. **7**: p. 205-229.

12. Willingham, C. and A. Rutherford, *Use of Osmium-Thiocarbohydrazide-Osmium and Ferrocyanide-reduced Methods Contrast and Preservation in to Enhance Membrane Contrast and Preservation in Cultured Cells.* The journal of histochemistry and cytochemistry, 1984. **32**: p. 455-460.
13. Litman, R.B. and R.J. Barrnett, *The mechanism of the fixation of tissue components by osmium tetroxide via hydrogen bonding.* Journal of Ultrasructure Research, 1972. **38**: p. 63-86.
14. Neiss, W.F., *Electron staining of the cell surface coat by osmium-low ferrocyanide *.* Histochemistry, 1984: p. 231-242.
15. Korn, E.D., *A chromatographic and spectrophotometric study of the products of the reaction of osmium tetroxide with unsaturated lipids.* The Journal of cell biology, 1967. **34**: p. 627-638.
16. Hayat, M.A., *Fixation for Electron Microscopy*. 1981: Academic Press.
17. Porter, K.R. and F. Kallman, *The properties and effects of osmium tetroxide as a tissue fixative with special reference to its use for electron microscopy.* Experimental Cell Research, 1953. **4**: p. 127-141.
18. Collinson, S.R. and M. Schroeder, *Osmium : Inorganic & Coordination Chemistry*, in *Encyclopedia of Inorganic and Bioinorganic Chemistry*. 2011.
19. Griffith, W.P., *Cyanide complexes of the transition metals.* Quarterly Reviews, Chemical Society, 1962. **16**: p. 188-207.
20. Opekar, F. and P. Beran, *Voltammetric study of the cyanide complexes of osmium-II. The behaviour of lower oxidation state cyanide complexes of osmium on a platinum rde.* Electrochimica Acta, 1977. **22**: p. 249-254.
21. Opekar, F. and P. Beran, *STUDY OF THE CYANIDE COMPLEXES THE REDUCTION THE CYANIDE COMPLEX OF Os (V1) ON A PLATINUM rde.* Electrochimica Acta, 1977. **22**: p. 243-248.
22. Tirler, A.O., et al., *Is the Hexacyanoferrate(II) Anion Stable in Aqueous Solution? A Combined Theoretical and Experimental Study.* Inorganic Chemistry, 2015. **54**: p. 10335-10341.
