# Supplementary material for: High-contrast en bloc staining of mouse whole-brain and human brain samples for EM-based connectomics
Source: Nat Methods. 2023 May 8;20(6):836–40. doi: 10.1038/s41592-023-01866-3 (PMC10250191; doi:10.1038/s41592-023-01866-3)
Supplement: Supplementary file 2 — Reporting Summary [file 41592_2023_1866_MOESM2_ESM.pdf]

nature research

Corresponding author(s): Moritz Helmstaedter, Kun Song

Last updated by author(s): Jan 18, 2023

Reporting Summary

Nature Research wishes to improve the reproducibility of the work that we publish. This form provides structure for consistency and transparency in reporting. For further information on Nature Research policies, see our Editorial Policies and the Editorial Policy Checklist.

Statistics

For all statistical analyses, confirm that the following items are present in the figure legend, table legend, main text, or Methods section.

n/a	Confirmed	
☐	☒	The exact sample size (*n*) for each experimental group/condition, given as a discrete number and unit of measurement
☐	☒	A statement on whether measurements were taken from distinct samples or whether the same sample was measured repeatedly
☐	☒	The statistical test(s) used AND whether they are one- or two-sided<br>*Only common tests should be described solely by name; describe more complex techniques in the Methods section.*
☒	☐	A description of all covariates tested
☐	☒	A description of any assumptions or corrections, such as tests of normality and adjustment for multiple comparisons
☐	☒	A full description of the statistical parameters including central tendency (e.g. means) or other basic estimates (e.g. regression coefficient) AND variation (e.g. standard deviation) or associated estimates of uncertainty (e.g. confidence intervals)
☐	☒	For null hypothesis testing, the test statistic (e.g. F, t, r) with confidence intervals, effect sizes, degrees of freedom and P value noted<br>*Give P values as exact values whenever suitable.*
☒	☐	For Bayesian analysis, information on the choice of priors and Markov chain Monte Carlo settings
☒	☐	For hierarchical and complex designs, identification of the appropriate level for tests and full reporting of outcomes
☒	☐	Estimates of effect sizes (e.g. Cohen's *d*, Pearson's *r*), indicating how they were calculated

Our web collection on statistics for biologists contains articles on many of the points above.

Software and code

Policy information about availability of computer code

Data collection	xT microscope (ThermoFisher Quanta), Scout-and-Scan (16.1.14271.44713 Zeiss XRM Versa 510)
Data analysis	TXM3DViewer (16.1.14271.44713 Zeiss), ImageJ (1.53q), GraphPad Prism 9, Matlab R2018a

For manuscripts utilizing custom algorithms or software that are central to the research but not yet described in published literature, software must be made available to editors and reviewers. We strongly encourage code deposition in a community repository (e.g. GitHub). See the Nature Research guidelines for submitting code & software for further information.

Data

Policy information about availability of data

All manuscripts must include a data availability statement. This statement should provide the following information, where applicable:

- Accession codes, unique identifiers, or web links for publicly available datasets
- A list of figures that have associated raw data
- A description of any restrictions on data availability

The source data for all the figures are shared via Edmond: https://doi.org/10.17617/3.RG58DU.

Field-specific reporting

Please select the one below that is the best fit for your research. If you are not sure, read the appropriate sections before making your selection.

☒ Life sciences ☐ Behavioural & social sciences ☐ Ecological, evolutionary & environmental sciences

For a reference copy of the document with all sections, see nature.com/documents/nr-reporting-summary-flat.pdf

Life sciences study design

All studies must disclose on these points even when the disclosure is negative.

Sample size	In most of our staining experiments, the changes to the experimental conditions (e.g., chemical type, temperature, incubation length) usually caused qualitatively different results (e.g. gradient vs. no gradient, breakage vs. no breakage etc.), which were also highly replicable. Thus, we typically have in each condition 3-4 samples. For the staining experiments on 2 mm and human samples (see Suppl. Table 3), number of replications was ≥3; for hemisphere and whole brain staining experiments, number of replications was ≥2 (Suppl. Table 3).
Data exclusions	no data were excluded
Replication	The final protocols for 2-3mm samples, mouse hemispheres and whole brain were replicated >10, 5 and 2 times, respectively.
Randomization	For the staining experiments with 2 mm samples, we made sure that the sample numbers are balanced across different locations (e.g. cortex vs. subcortical region). For all staining experiments, we made sure that we were using mice with similar age (1-3 months old) and gender (mostly male) and strain (C57BL6/J).
Blinding	The effect size (successful staining vs samples not amenable to low-vaccum EM imaging) was so large that bias of the observer was not a relevant concern.

Reporting for specific materials, systems and methods

We require information from authors about some types of materials, experimental systems and methods used in many studies. Here, indicate whether each material, system or method listed is relevant to your study. If you are not sure if a list item applies to your research, read the appropriate section before selecting a response.

Materials & experimental systems

n/a	Involved in the study	
☒	☐	Antibodies
☒	☐	Eukaryotic cell lines
☒	☐	Palaeontology and archaeology
☐	☒	Animals and other organisms
☐	☒	Human research participants
☒	☐	Clinical data
☒	☐	Dual use research of concern

Methods

n/a	Involved in the study	
☒	☐	ChIP-seq
☒	☐	Flow cytometry
☒	☐	MRI-based neuroimaging

Animals and other organisms

Policy information about studies involving animals; ARRIVE guidelines recommended for reporting animal research

Laboratory animals	Adult C57BL6/J mice (male/female, P30-P90). Housing conditions: room temperature 22°C, relative humidity 55% (+/- 10%) , light cycle 12 hrs light/12 hrs dark. Animals are kept in breeding in type 2 long IVC greenline cages with red house and nesting material, bedding Lignocel BK8-15.<br>Food: Autoclaved water and food "Sniff standard mouse extruded breeding or mouse/rat husbandry" both ad libitum.
Wild animals	No wild animals were used in this study.
Field-collected samples	No field-collected samples were used in this study.
Ethics oversight	Regierungspräsidium Darmstadt, Germany, approvals V54 - 19 c 20/15 - F126/1028 and F126/1002

Note that full information on the approval of the study protocol must also be provided in the manuscript.

Human research participants

Policy information about studies involving human research participants

Population characteristics	Neurosurgical patient who consented to usage of cortical access tissue for research. 69-year old female patient.
Recruitment	Recruitment by neurosurgeon based on type of planned operation. Possible bias is selection of patient by neurosurgeon and willingness of patient to consent. Since no patient performance was relevant as observable, these biases are considered not relevant.
Ethics oversight	Ethics Committee of the Technical University of Munich School of Medicine (Ethikvotum 184/16S and 273/21 S-EB). All patients had given their written informed consent.

Note that full information on the approval of the study protocol must also be provided in the manuscript.
